# The association between diabetes mellitus and low back pain: a systematic review and meta-analysis

**DOI:** 10.1186/s12891-026-10226-z

**Published:** 2026-07-14

**Authors:** Muje El Noaimi, Niyaz Hareni, Lars Jehpsson, Björn E. Rosengren

**Affiliations:** 1 Departments of Clinical Sciences and Orthopedics, Lund University, Skåne University Hospital, Malmö, Sweden; 2 Department of Orthopedics, Halland Hospital, Halland, Sweden

**Keywords:** Low back pain, Degenerative spine disease, Diabetes, Prevalence, Incidence

## Abstract

**Study design:**

Systematic review and meta-analysis.

**Background and objective:**

Both diabetes mellitus (DM) and low back pain (LBP) are leading global causes of disability, and emerging evidence suggests a link between them. Understanding the DM-LBP association is clinically important as it may facilitate more integrated treatment strategies; however, the exact nature of this relationship remains poorly evaluated. This systematic review and meta-analysis aimed to compare the comorbid prevalence of LBP and DM and to evaluate their longitudinal relationship in adult populations.

**Methods:**

A systematic search was conducted across PubMed, EMBASE, CINAHL and Google scholar from inception to October 2024. This review included English-language studies involving adults (≥ 18 years) with DM (type 1, type 2, or prediabetes) and LBP. Eligible study designs included cohort studies, randomized controlled trials, case-control, twin, and cross-sectional studies.

Exclusion criteria applied to review articles, single case studies and conditions including gestational diabetes, fractures, malignancies, infections, and post-traumatic- or rheumatoid arthritis. Two independent reviewers performed study screening, data extraction, and methodological quality assessment using Newcastle-Ottawa Scale. Random-effects meta-analysis and regressions were performed using R software. Primary outcomes centered on the prevalence and longitudinal incidence of LBP and DM across diverse global populations.

**Results:**

Twenty-six studies (28 datasets) involving 1,359,721 participants from 17 countries were included, comprising 20 cross-sectional, 4 cohort and 2 case-control designs, with a predominant representation from Asia (62%). Methodological quality assessment (via Newcastle-Ottawa Scale) classified 28.6% of datasets as low risk of bias (Good), 46.4% as moderate risk (Fair), and the remainder as high risk of bias (Poor).

The pooled LBP prevalence difference was 8.7 percentage points higher in the diabetic population compared to the non-diabetic population (95% CI: 4.4–13). In contrast, the pooled DM prevalence difference was 7.4 percentage points higher in the LBP population compared to the non-LBP population (95% CI: 3.8–11). The pooled odds ratio (OR) for LBP in patients with DM, compared with those without DM, was 1.66 (95% CI: 1.34–2.06). Univariate meta-regression model identified mean age as a significant moderator (p = 0.033).

Utilizing adjusted risk estimates, the longitudinal analysis of the overall risk of LBP in patients with DM was non-significant (RR 1.22; 95% CI: 0.91–1.62). A sex difference was however observed (p = 0.037) where men had a reduced risk (RR 0.83; 95% CI: 0.71–0.96) and women possibly an increased risk (RR 1.42; 95% CI: 0.88–2.32). Conversely, LBP was modestly associated with an increased risk of developing DM (RR 1.15; 95% CI: 1.02–1.29), though this association was slightly stronger in women. (RR 1.31; 95% CI: 1.11–1.56).

**Discussion:**

The certainty of evidence for all outcomes was very low. Evidence was limited by large statistical heterogeneity (I2 > 95%), potential publication bias, and clinical indirectness arising from non-standardized definitions of LBP. Thus, while this review highlights a potential bidirectional association between diabetes and low back pain, the findings must be interpreted with caution. Limited longitudinal data also suggest that LBP was modestly associated (15%) with a higher risk of developing DM compared to non-LBP peers – potentially pointing to a stronger association in women (31%). These findings may be considered in the clinical setting for both patients with DM and for those with LBP. The low certainty of evidence highlights the critical need for more standardized, high-quality longitudinal research.

**Registration and funding:**

The review as registered in PROSPERO (ID: CRD42025643242) and the primary funding was received from non-profit grant givers (ALF, FoUU and Skåne University hospital, Lars Holmqvist, and Sparbanksstiftelsen Varberg funds).

**Supplementary Information:**

The online version contains supplementary material available at 10.1186/s12891-026-10226-z.

## Introduction

Diabetes mellitus (DM) is a chronic metabolic disease, characterized by elevated blood glucose levels due to inadequate insulin secretion, impaired insulin action or a combination of both [1, 2]. The condition has become a global pandemic affecting more than half a billion people worldwide and is projected to increase by 45%, reaching 853 million by 2050 [3]. DM stands as the eighth leading cause of death and disability globally [4]. In parallel, low back pain (LBP) is the principal reason for years lived with disability (YLDs) and affects 619 million individuals across the globe. By 2050, it is estimated that 843 million people will suffer from LBP [5].

While LBP and DM are different conditions, they may clinically manifest with similar sensory disturbance and radiating pain, which complicates clinical diagnosis and patient management [6, 7]. They also share several common risk factors such as obesity, age and smoking [5, 8, 9], and both are known to reduce quality of life [10–12]. Consequently, establishing a definitive relationship remains challenging due to overlapping risk factors and the potential for residual confounding [8, 9, 11].

Previous research suggests a link between DM and LBP where elevated average blood sugar levels may be an underrecognized determinant of LBP [13]. People with DM also appear to be at higher risk of developing musculoskeletal pain, including low back pain [14, 15]. Furthermore, in LBP patients the co-occurrence of DM has been associated with an increased use of analgesics [16]. While the underlying pathophysiological relationships are not fully understood, biochemical investigations have shown that high blood glucose is associated with disc degeneration, reduced vertebral bone mass, and increased catabolic reactions in the intervertebral discs, which can in turn precipitate low back pain [17–19].

Understanding the DM-LBP association is clinically important for patients, clinicians, and researchers, as this may facilitate new or more integrated treatment strategies. This systematic review and meta-analysis aimed to compare the comorbid prevalence of LBP and DM and to evaluate their longitudinal relationship in adult populations.

## Methods

This systematic review was conducted according to the Preferred Reporting Items for Systematic Reviews and Meta-Analysis (PRISMA) guidelines [20] and was registered in PROSPERO (Registration ID: CRD42025643242) (S1 Text).

### Aim

#### Primary research questions (RQ)

- RQ1: What is the prevalence of LBP in people with DM compared to non-diabetic peers?
- RQ2: What is the prevalence of DM in people with LBP compared to non-LBP peers?

#### Secondary RQ (SRQ)

- SRQ1: What is the association between LBP and DM?
- SRQ2: Is there any longitudinal relationship between LBP and DM?

### Search strategy and data sources

**Fig. 1:**
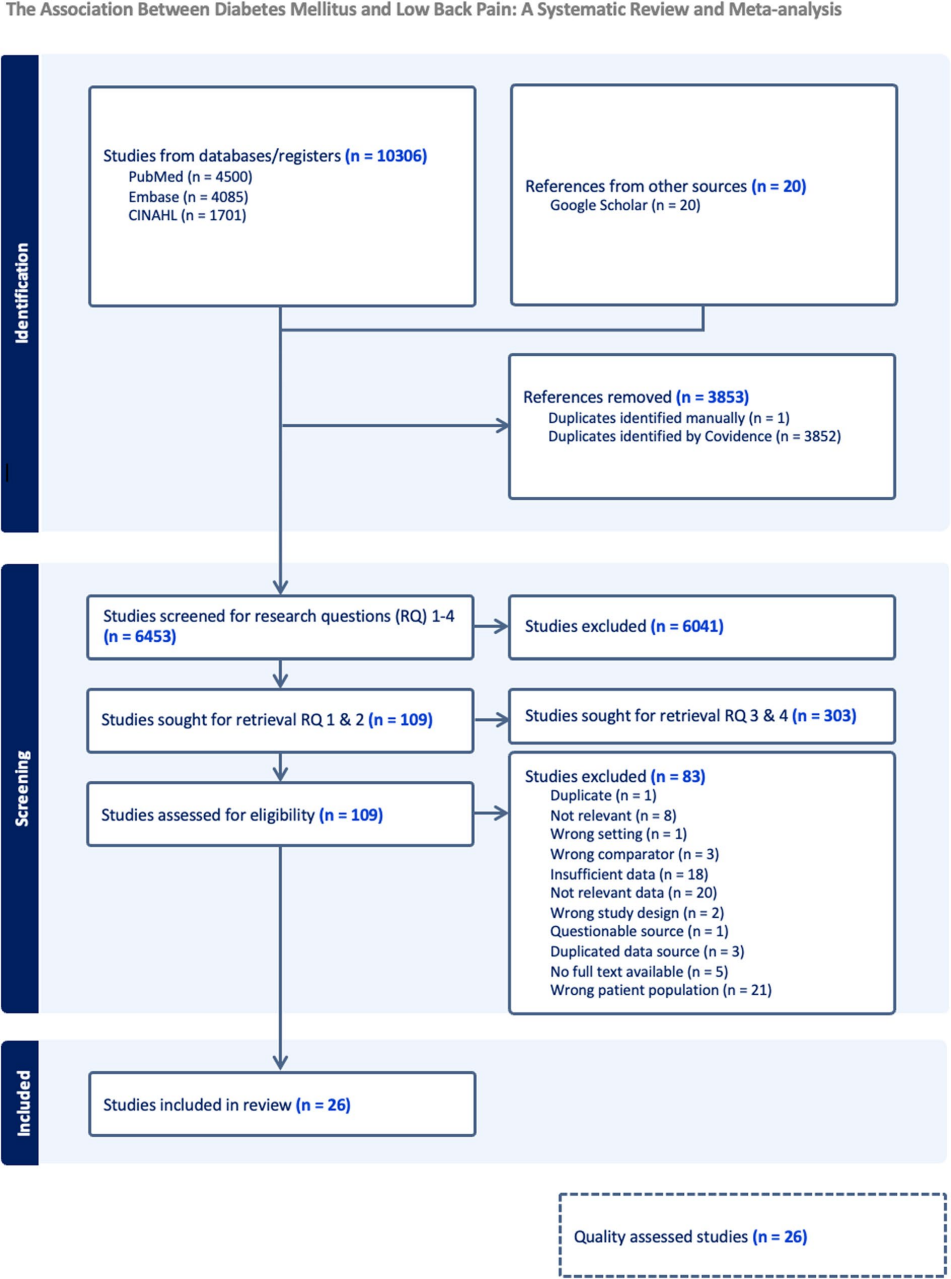
Search results (PRISMA flowchart)

The search criteria and strategy were developed together with an information specialist (MB). The search strategy was initially designed to address four RQs (Fig. 1). However, this current review focuses exclusively on RQ 1 and RQ 2 with its secondary questions listed in the Aims section above.

A systematic search of PubMed, EMBASE, CINAHL was conducted from inception to October 2024. The search was restricted to English-language publications from 2000 onwards. A combination of Medical Subject Headings (MeSH) and free-text keywords related to both exposure and outcome (LBP and/or DM) were used. Representative MeSH terms for diabetes conditions included ‘Diabetes Mellitus, Type 1’, ‘Diabetes Mellitus, Type 2’, ‘Prediabetic’ and ‘Diabetes Complications’. For spinal pathology, MeSH terms such as ‘Low Back Pain’, ‘Intervertebral Disc Hernia’, ‘Spinal Stenosis’, ‘Spondylolisthesis’, and ‘Sciatica’ were applied. The search was also supplemented by specific keywords and synonyms (e.g., ‘Glucose intolerance, ‘disc herniation’, ‘degenerative disc disease’) across all databases. No restrictions were placed on the study setting (e.g., community-based, hospital-based or primary care) or geographic location. To ensure a comprehensive search, an extensive range of terms and Boolean operators were used. The full detailed strategy is provided in S2 Text (Search strategy).

Additionally, Google Scholar was searched during the same search period to identify grey literature, using the same keywords, publication data constraint, and language filters as in the primary databases. The results in Google Scholar were sorted by relevance and screened until no further eligible studies were identified. Identified records were managed in Endnote software and exported to Covidence systematic review software for screening and selection processes [21].

### Eligibility criteria

The inclusion and exclusion criteria followed the PICOS framework (S3 Text; Table 2). Eligible studies included (i) adults (≥ 18 years) with type 1 or type 2 DM (or similar forms of diabetes) and low back pain (or spinal diagnosis causing LBP); (ii) prediabetic people with low back pain (or spinal diagnosis causing LBP).

Cohort studies, randomized controlled trials (RCT), case-control studies, twin and cross-sectional studies were included.

Studies were excluded if they: (i) included individuals with gestational diabetes or (ii) people with other spine pathology such as fractures, cancers, infections, post-traumatic- and rheumatoid arthritis; (iii) were non-English publications, reviews, single case studies or animal studies.

World Health Organization (WHO) defines low back pain as pain between the lower edge of the ribs and buttock [22]. Studies were included if the authors specifically described the study population having LBP symptoms or a symptomatic lumbar spine disease. If the studies included multiple regions of the back, only data specific to the lower back were included in this review.

The primary outcome of interest was prevalence, while incidence was considered a secondary outcome.

### Study selection and screening process

The search results were imported into Covidence systematic review management software [21]. Duplicates were removed and reported into the PRISMA flow diagram (Fig. 1). One reviewer (ME) screened the titles and abstracts for eligibility according to the inclusion and exclusion criteria. Uncertainties were resolved through consultation with another reviewer (BR). Full-text screening was performed by two independent reviewers (ME and NH). Disagreements were resolved through consensus or third-party arbitration (BR). Reasons for the excluded studies were recorded. Inter-rater reliability was assessed using Cohen’s Kappa (𝜅), with values interpreted per McHugh (2012): 0.21–0.39 (minimal), 0.40–0–59 (weak), 0.60–0.79 (moderate), 0.80–0.90 (strong), and > 0.90 (almost perfect) [23]. To identify additional relevant studies, reference tracking was performed during the screening process.

### Data extraction

The research team manually created the data extraction form using Microsoft Word. Data extraction was initially performed by one reviewer (ME) and subsequently reviewed and verified by a second reviewer (NH). Any disagreements were resolved through discussion, or third-party arbitration (BR).

The following extracted data were included:

- Descriptive details – author, country, continent and year of publication.
- Study design, setting and whether the study was population-based or not.
- Study sample, including the diabetic population.
- Demographics – mean age and body mass index (BMI) and sex distribution in percentage.
- Assessment of DM and LBP type.

*(LBP definition was categorized as Chronic if duration ≥ 3 months or Other if under 3 months*,* not defined*,* or not in line with the definition of chronic LBP)*

- Prevalence/incidence data.
- Data extraction comments.

### Risk of bias assessment and certainty of evidence

Two reviewers (ME and NH) independently assessed the risk of bias using the Newcastle-Ottawa Scale (NOS) for cohort and case-control studies [24]. NOS is a tool identified by the Cochrane Handbook for the assessment of non-randomized studies [25]. For cross-sectional studies, a modified version of the NOS was applied [26]. Any disagreements were resolved through discussion or arbitration by a third reviewer (BR).

NOS is a traditional star-based system, and a domain-based approach was adopted rather than relying on aggregate star counts. To align with the Agency for Healthcare Research and Quality (AHRQ) standards [27], the NOS scores were converted into Good, Fair, and Poor-quality rankings based on specific thresholds across three different domains: Selection, Comparability and Exposure/Outcome (S3 text). A Good rating required 3–4 stars in Selection (4–5 for cross-sectional studies), 1–2 in Comparability (2–3 for cross-sectional studies) y, and 2–3 in Exposure/Outcome (all studies). A Fair rating required 2 stars in Selection (2–3 for cross-sectional studies, 1–2 in Comparability (2–3 for cross-sectional studies) y, and 2–3 in Exposure/Outcome (all studies). A Poor rating was assigned if a study scored 0–1 in Selection, 0 in Comparability (0–1 for cross-sectional studies), or 0–1 in Outcome (all studies).

For visual synthesis, the quality ratings were mapped into a domain-based traffic light plot. The AHRQ classifications were translated into categorical risk of bias judgement. Good quality was categorized as low risk (green), Fair as some concerns (yellow), and Poor as high risk (red). To ensure that the visual plot remained consistent with the AHRQ framework, the overall judgement followed the weakest link principle, where the highest risk identified in any single domain determined the study’s final rating. Visual synthesis of the risk of bias findings was performed using the robvis tool [28]. Full star-by-star breakdown for each included study is provided in S3 text (Tables 3 and 4).

To ensure consistency, study design was reviewed and categorized by the reviewers according to methodological characteristics, including data structures and analytical directions.

The certainty of the evidence for the association between DM and LBP was assessed using the GRADE approach [29]. Two reviewers (ME and NH) independently evaluated the evidence across five domains: risk of bias, inconsistency, indirectness, imprecision, and publication bias. As the included studies were observational, the evidence certainty started at low and was subsequently downgraded based on the five domains. The certainty was categorized as high, moderate, low, or very low. Any disagreements were resolved through discussion or arbitration by a third reviewer (BR).

### Data analysis

Data management was conducted in Microsoft Excel while all statistical analyses performed in R Statistical Software (v4.5.2) [30] with Meta package. Random-effects model was employed for all analyses. Pooled prevalences were calculated using Generalized Linear Mixed Models with logit transformation. Between-group comparisons were assessed as Prevalence Differences per 100 observations using an inverse-variance random model. Variance was estimated using the maximum-likelihood estimator. For the longitudinal association, adjusted effect sizes were pooled using the generic inverse-variance method.

Wald test was used to assess the statistical significance of all pooled effect sizes and meta-regression coefficients. Results were considered statistically significant if the 95% confidence interval did not cross the null value (0 for prevalence difference; 1.0 for ratios). Studies restricted to 100% baseline prevalence of either DM or LBP, or if a non-exposed or unaffected control group was unavailable, were excluded from the comparative analysis due to statistical ‘ceiling effect’. To interpret the magnitude of the associations, effect sizes of 1.5, 3.0, and 5.0 were used to define small, medium and large impacts, respectively [31]. These thresholds were justified by adapting the effect size conversion framework proposed by Chen et al., (2010). Heterogeneity was evaluated using the *I*^*2*^ statistic and categorized as low (< 25%), moderate (25–75%) or high (> 75%). To explore the sources of heterogeneity, subgroup- and sensitivity analyses were performed. For the assessment of the influence of age, BMI and female gender on the pooled estimates, a random-effects meta-regression was utilized. Publication bias was screened using Egger’s regression.

### Protocol deviations

In accordance with PRISMA 2020 guidelines, a deviation from the original research protocol is reported. The initial literature search included terms for surgical and non-surgical treatment outcomes (previously intended as RQ3 and RQ4). These objectives were ultimately excluded to prioritize a focused synthesis on the primary and secondary aims outlined in this review. The selection process and exclusions are transparently documented in the PRISMA flow chart (Fig. 1). Furthermore, following peer-review feedback, the quantitative analyses expanded to include a more comprehensive descriptive synthesis for RQ1 and RQ2. This was followed by meta-analysis for SRQ1 and evaluation of the longitudinal association for SRQ2.

## Results

### Literature selection

The search identified 10,326 records (PubMed: 4,500; Embase: 4,085; CINAHL: 1,701; Google Scholar: 20). After removing duplicates and screening titles and abstracts, 109 articles were selected for full-text review. Inter-rater reliability for the selection process was substantial, with an observed agreement of 89% (Cohen’s = 0.70; 95% CI: 0.55–0.86). A total of 26 studies were included in the final review [15], [32–56]. The most frequent reason for exclusion was ineligible patient population. The screening process and specific reasons for exclusion are detailed in the PRISMA flow diagram (Fig. 1).

### Study characteristics

Studies reported data from 17 countries across four continents [15], [32–56], with a predominant representation from Asia (62%; Table 1, S3 text: Fig. 4) [15, 32–34, 38–40, 44, 47–50, 52, 54–56]. The literature comprised 20 cross-sectional [32–35, 37, 38, 40–51, 53, 55, 56], four cohort [36, 39, 46, 53] and two case-control designs [52, 54]. Notably, two studies provided both cross-sectional and longitudinal datasets [46, 53](Table 1).

Twelve studies were population-based [34–36, 41, 45, 46, 50, 51, 53–56] (S3 text: Fig. 5). The average study duration for cross-sectional studies was 1.8 years (range: 0.25–4.5), while case-control studies averaged 10.5 years (range: 4–17). For cohort studies, the mean follow-up period was 9 years (range 2–13).

The total sample comprised 1,359,721 participants (range: 141–959,360), of whom 593,961 (44%) had diabetes (Type 2 DM or unspecified; range: 11–479,680). The pooled mean BMI and age for the study population were 27.2 *kg/m²* (range: 23.3–31.3) and 52 years (range: 26.3–66.3), respectively (Table 1; S3 text: Figures 6 and 7). The majority of the sample were women (mean 57.7%; range: 34.1–78.9; Table 1).

**Table 1:** Summary of study characteristics, participant demographics, and clinical definitions of DM and LBP

Author (Year)	Country (Continent)	Study Design/Length (years)	Population-based	Demographics Age (yrs) BMI (kg/m2) Sex (% Female)	Total *N* (DM *N*)	LBP definition	DM type	Quality	Data extraction comments
Abbas et al. (2013) [52]	Israel (Asia)	CC/4,0	No	A: 63.2 B: 28.7 S: 50.7% F	345 (104)	Chronic	Unspecified	Good	Means calculated
Alsubaie et al. (2024) [32]	Saudi Arabia (Asia)	CS/1,0	No	A: 33.0 B: 27.0 S: 61.0% F	2 181 (116)	Other	Unspecified	Poor	
Chang et al. (2022) [33]	China (Asia)	CS/1,0	No	A: 37.5 B: 25.9 S: 41.7% F	530 (18)	Other	Unspecified	Fair	
Ha et al. (2014) [34]	Republic of Korea (Asia)	CS/3,0	Yes	A: 48.1 B: NA S: 56.4% F	13 299 (3 685)	Chronic	Unspecified	Fair	Impaired fasting glucose group included
Hassoon et al. (2017) [35]	USA (North America)	CS/2,0	Yes	A: 43.0 B: NA S: 50.8% F	5 106 (515)	Chronic	Unspecified	Fair	
Jacob et al. (2021) [36]	Germany (Europe)	RC/10,0 (follow up)	Yes	A: 62.5 B: NA S: 58.0% F	139 002 (69 501)	Other	T2DM	Good	
KaracIF et al. (2022) [37]	Turkey (Europe)	CS/1,5	No	A: 49.4 B: 29.0 S: 75.2% F	141 (23)	Chronic	Unspecified	Poor	
Anekstein et al. (2010) [38]	Israel (Asia)	CS/1,5	No	A: 59.5 B: NA S: 53.6% F	349 (78)	Chronic	Unspecified	Fair	Means of age groups. Osteoporotic fracture group excluded.
Wang et al. (2021) [39]	Taiwan (Asia)	RC/13,0 (follow up)	No	A: 26.3 B: NA S: 58.8% F	10 470 (173)	Other	Unspecified	Good	
Ibrahim et al. (2022) [40]	Malaysia (Asia)	CS/NA	No	A: NA B: NA S: 67.1% F	161 (89)	Other	T2DM	Poor	Only data from 12-months groups
Molsted el al. (2012) [41]	Denmark (Europe)	CS/4,5	Yes	A: 60.5 B: 31.3 S: 52.0% F	3 874 (951)	Other	T2DM	Poor	Age and BMI means calculated
Olaosebikan et al. (2019) [42]	Nigeria (Africa)	CS/0,5	No	A: 58.4 B: NA S: 78.9% F	536 (268)	Chronic	T2DM	Good	Age mean calculated
Abaraogu et al. (2017) [43]	Nigeria (Africa)	CS/NA	No	A: 53.1 B: 26.5 S: 55.0% F	347 (167)	Chronic	T2DM	Good	Age and BMI means calculated
Asadian et al. (2016) [44]	Iran (Asia)	CS/2,0	No	A: 48.3 B: NA S: 70.9% F	330 (50)	Chronic	Unspecified	Fair	Age mean calculated
de Luca et al. (2023) [45]	USA (North America)	CS/2,0	Yes	A: NA B: NA S: 51.1% F	26 926 (2 881)	Chronic	Unspecified	Fair	Data from LBP group only
Eivazi et al. (2012) [15]	Iran (Asia)	CS/0,3	No	A: 51.0 B: 27.3 S: 73.4% F	417 (317)	Other	T2DM	Poor	Age and BMI means calculated
Heuch et al. (2018) [46]	Norway (Europe)	CS/2,0	Yes	A: NA B: NA S: 52.2% F	45 157 (974)	Chronic	Unspecified	Fair	
		PC/11,0 (follow up)		A: NA B: NA S: 54.5% F	30 380 (1 202)			Good	
Iizuka et al. (2017) [47]	Japan (Asia)	CS/1,0	No	A: 63.8 B: 23.6 S: 64.8% F	193 (11)	Chronic	Unspecified	Fair	Age and BMI means calculated. Data from non-specific LBP only.
Al-Rudaini et al. (2022) [48]	Oman (Asia)	CS/1,0	No	A: 55.4 B: 30.2 S: 67.5% F	200 (200)	Chronic	T2DM	Poor	
Jena et al. (2022) [49]	India (Asia)	CS/0,5	No	A: 53.6 B: 25.8 S: 34.1% F	370 (370)	Other	T2DM	Fair	Data from LBP group only
Maeda et al. (2018) [50]	Japan (Asia)	CS/2,0	Yes	A: 66.3 B: 23.3 S: 67.0% F	968 (80)	Other	T2DM	Fair	
Real et al. (2019) [51]	USA (North America)	CS/5,0	Yes	A: 49.0 B: 28.3 S: 52.0% F	11 756 (1 015)	Other	Unspecified	Fair	
Dario et al. (2017) [53]	Spain (Europe)	PC/3,0(follow up)	Yes	A: 56.7 B: 27.2 S: 55.0% F	1 612 (210)	Chronic	T2DM	Poor	Mean follow-up time
		CS/2,0		A: 53.6 B: 27.4 S: 55.0% F	2 096 (229)			Fair	
Shemesh et al. (2023)	Israel (Asia)	CC/17,0	Yes	A: NA B: NA S: 50.0% F	99 130 (30 443)	Chronic	Unspecified	Good	
Uesugi et al. (2013) [55]	Japan (Asia)	CS/1,0	Yes	A: NA B: NA S: 54.7% F	4 485 (529)	Other	Unspecified	Fair	Data from DM diagnosed group only
Park et al. (2021) [56]	Republic of Korea (Asia)	CS/2,0	Yes	A: NA B: NA S: 49.8% F	959 360 (479 680)	Other	T2DM	Good	Data from LDD group only.

Participant demographics (Age and BMI) are reported as means. A: Age (yrs); B: BMI (); S: Sex (% Female)

Abbreviations: CC: Case-control; CS: Cross-sectional; DDD: Degenerative disc disease; DLSS: Degenerative lumbar spinal stenosis; DM: Diabetes Mellitus; NA: Not Available (due to missing data or non-continuous variables); PC: Prospective cohort; RC: Retrospective cohort; T2DM: Type 2 Diabetes Mellitus; LDD: Lumbar disc degeneration/disease

### Low back pain and diabetes mellitus assessment

Assessment methods for LBP and DM varied across the 28 included datasets (Table 1). Sixteen datasets provided sufficient information to classify the population with chronic LBP [34, 35, 37, 38, 42–48, 52–54]. The LBP in the population from the remaining datasets were categorized as Other due to shorter or unspecified durations. Regarding diabetes classification, only data from 12 datasets were sufficient to categorize the population as having Type 2 DM (T2DM) [15, 36, 40–43, 48–50, 53, 56]. The remainder were classified as Unspecified. No studies reported Type 1 DM (Table 1).

### Methodological quality

**Fig. 2:**
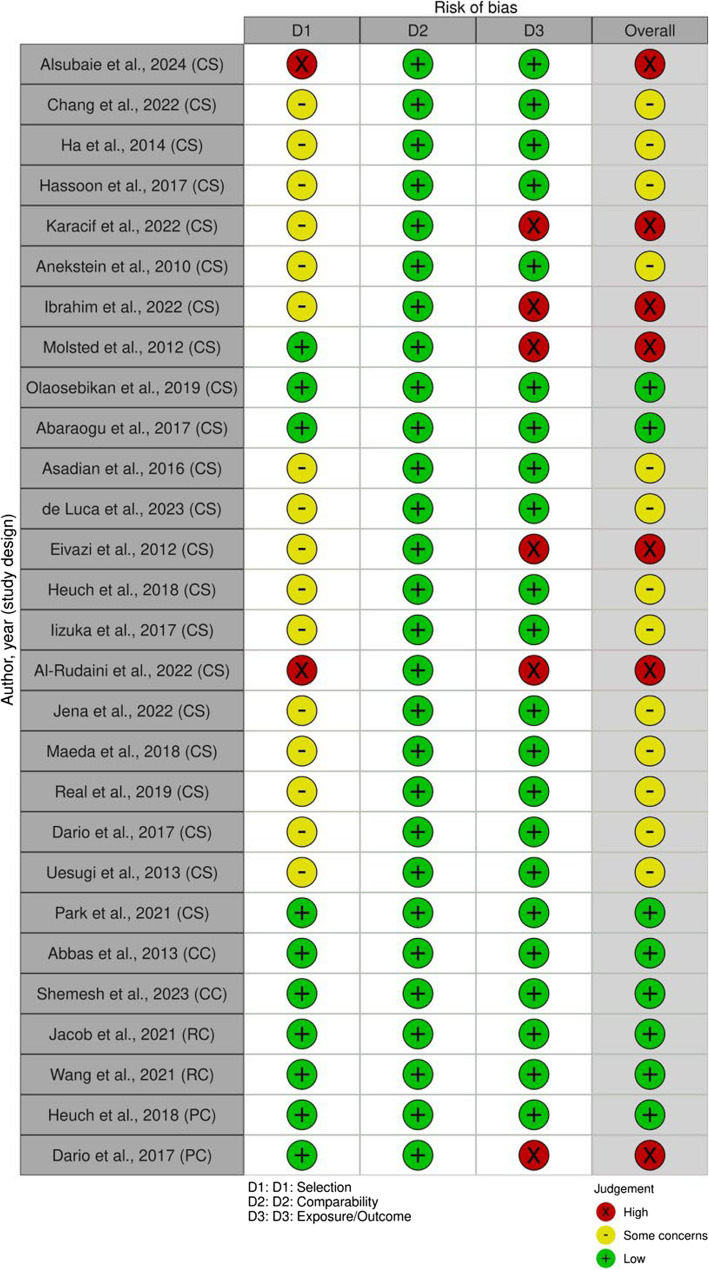
Traffic-light plot (risk of bias assessments). Note: Ratings for each study assessment across three domains: D1 (Selection), D2 (Comparability), and D3 (Outcome/Exposure). (+) indicates low risk, (-) indicates some concerns, and (x) indicates high risk. CS - Cross sectional design, CC – Case-control design, RC/PC – retrospective/prospective cohort design

Risk of bias was evaluated for 28 datasets (representing 26 studies), and the NOS scores were converted into Good, Fair, or Poor-quality rankings according to AHRQ standards (Table 1; S3 text: Table 4). The overall methodological quality is visualized in the traffic-light- (Fig. 2) and summary plot (S3 Text: Figures 8 and 9).

Regarding quality, eight datasets (28.6%) were rated as Good [36, 39]. [42, 43, 46, 52, 54, 56], thirteen (46.4%) as Fair [33–35, 38], [44–47], [49–51, 53, 55], whereas the remainder seven were rated as Poor [15, 32, 37, 40, 41, 48, 53].

Domain-specific analysis revealed that 100% of the datasets were rated as low risk of bias in the comparability domain. The majority of the case-control and cohort datasets demonstrated low risk of bias across all domains except one dataset that was rated as Poor (high risk of bias) due to deficiencies in the exposure/outcome domain [53]. The cross-sectional datasets showed a higher variability in the Selection domain, with nearly 50% rated as some concerns and two as high risk bias [32, 48] (Fig. 2; S3 text: Table 4). 

### Prevalence of LBP in DM vs. non-DM populations

For the analysis of LBP prevalence in DM populations, 18 cross-sectional datasets were included [15, 34, 35, 40–51, 53, 55, 56] and four were omitted from the pooled estimate due to invariant outcomes (ceiling effects) [32, 33, 37, 38]. The pooled LBP prevalence was 33.0% (95% CI: 23.8–43.8; *I*^2^ = 99.3%) in the DM population. Individual study estimates ranged from a minimum of 5.6% (95% CI: 3.17–9.06) [42] to a maximum of 78% (95% CI: 64–88.5) [44].

Regarding the analysis for the non-diabetic population, 15 datasets were included [15, 34, 35, 41–47, 50, 51, 53, 55, 56] and seven studies were excluded due to invariant outcomes (ceiling effects) [32, 33, 37, 38, 40, 48, 49]. The pooled LBP prevalence was 21.3% (95% CI: 14.1–31, *I*^2^ = 99.8%). Individual estimates ranged from 2.2% (95% CI: 0.83–4.8) [42] to 64.6% (95% CI: 58.7–70.2) [44].

Forest plots and individual study prevalence estimates are provided in S3 text (Figs. 10 and 11).

### Comparative analysis

Fifteen studies provided comparative data between diabetic and non-diabetic populations [15, 34, 35, 41–47, 50, 51, 53, 55, 56]. The pooled prevalence difference was 8.7% points higher in the diabetic population compared to the non-diabetic population (95% CI: 4.4–13; z = 3.98; *I*^2^ = 96.1%; S3 text: Fig. 12).

### Publication bias

Egger’s Regression test indicated asymmetry (*p* = 0.016; S3 text). Visual inspection of the contour-enhanced funnel plot showed that several studies were positioned within the areas of statistical significance (*p* < 0.05 and < 0.01; S3 text). A trim-and-fill sensitivity analysis was performed to account for potential small-study effects, resulting in an adjusted pooled prevalence difference of 2.36% (95% CI: −2.72–7.43; S3 text: Fig. 13).

### Prevalence of DM in LBP vs. non-LBP populations

For the analysis of DM prevalence in LBP population, 19 cross-sectional datasets were included [15, 32–35, 37, 38, 41–47, 50, 51, 53, 55, 56] and three studies were omitted due to invariant outcomes (ceiling effects) [40, 48, 49]. The pooled DM prevalence in the LBP population was 20.2% (95% CI: 12.2–31.3, *I*^2^ = 99.8%). Individual study estimate ranged from a minimum of 3.4% (95% CI: 2.0–5.3) [33] to a maximum of 81.1% (95% CI: 75.6–85.7; S3 text: Fig. 14) [15].

Regarding the non-LBP population, 15 datasets were included [15, 34, 35, 41–47, 50, 51, 53, 55, 56] and seven studies were omitted due to invariant outcomes (ceiling effects) [32, 33, 37, 38, 40, 48, 49]. The pooled DM prevalence was 15.9% (95% CI: 9.2–26.1; *I*^2^ = 100%). Individual estimates ranged from 2.1% (95% CI: 1.9–2.2)^46,^ to 68.6% (95% CI: 61.1–75.6; S3 text: Fig. 15) [15].

### Comparative analysis

Fifteen studies provided comparative data between LBP and non-LBP populations [15, 34, 35, 41–47, 50, 51, 53, 55, 56]. The pooled prevalence difference was 7.4% points higher in the LBP population compared to the non-LBP population (95% CI: 3.8–11.0; z = 4.01; *I*^2^ = 97.6%; Fig. 16).

### Publication bias

A symmetrical distribution of the studies was demonstrated in the funnel plot through visual inspection and confirmed by Egger’s test (*p* = 0.18). Further details and funnel plot visualization are provided in S3 text (Fig. 17).

### The association between DM and LBP

**Fig. 3:**
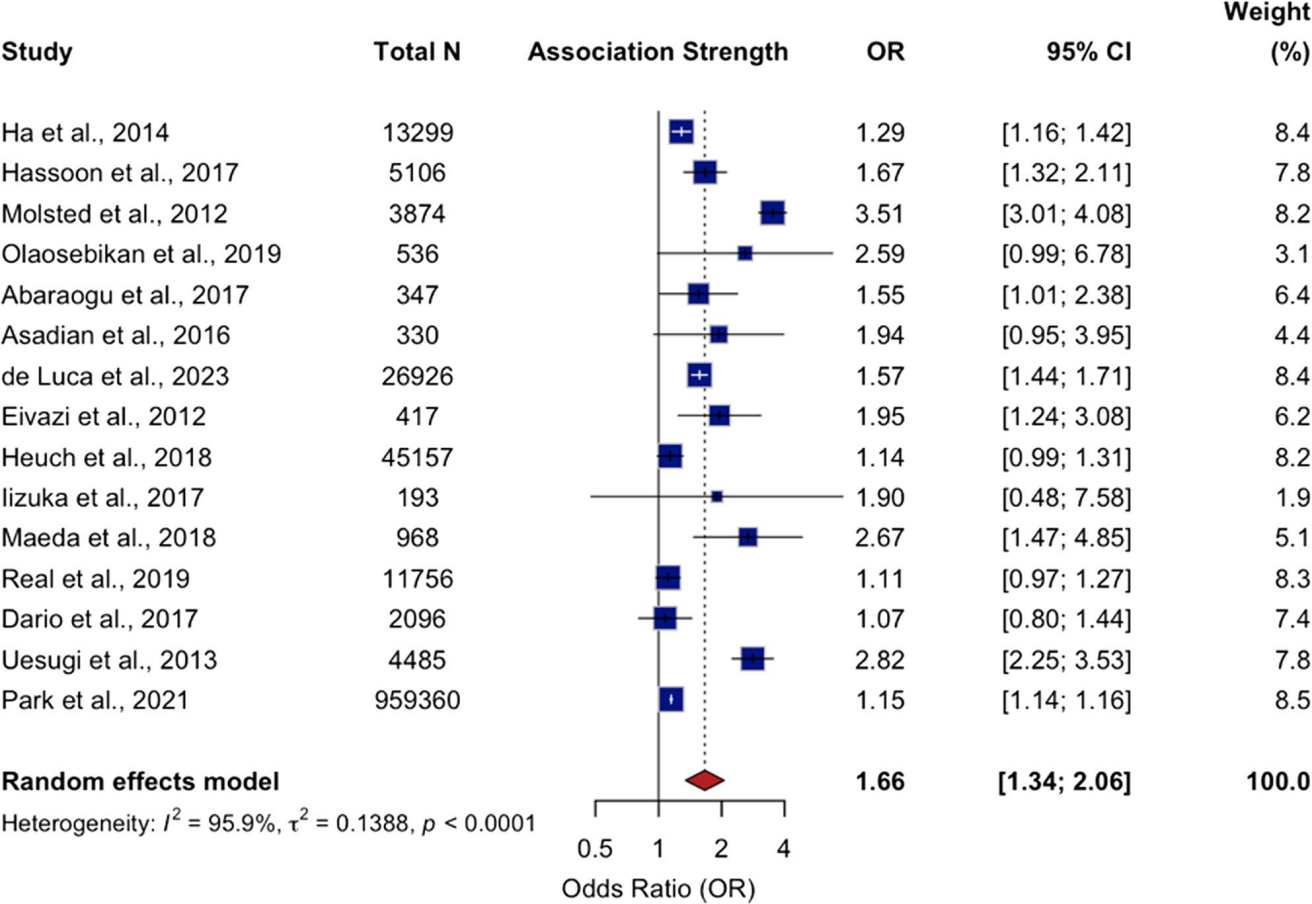
The pooled odds ratio (OR) for the association between DM and LBP, indicating that individuals with DM had 66% higher odds of suffering from LBP than individuals without DM

Fifteen cross-sectional studies provided data for a pooled analysis on the association of DM and LBP [15, 34, 35, 41–47, 50, 51, 53, 55, 56]. The pooled odds ratio (OR) for LBP in patients with DM, compared with those without DM, was 1.66 (*N* = 1,074,850; 95% CI: 1.34–2.06; *I*^2^ = 95.9%; Fig. 3).

### Publication bias

The assessment of publication bias for the association between DM and LBP revealed a significant funnel plot asymmetry (Egger’s test: *p* = 0.018). Visual inspection of the contour-enhanced funnel plot showed that most of the included studies were within the zones of statistical significance (*p* < 0.05 and < 0.01; S3 text). A trim-and-fill sensitivity analysis was conducted to address the observed asymmetry, which simulated seven hypothetical studies to account for suspected publication bias. Taking the simulation into account resulted in an adjusted pooled odds ratio of 1.21 (95% CI: 0.93–1.59). Further details and funnel plot visualization are provided in S3 text (Fig. 18).

### Subgroup analysis

While the direction of the effect was consistent with all the 15 studies, a significant heterogeneity (*I*^2^ = 95.9) was observed. To explore this, subgroup analyses were conducted based on geographical location, population-based studies, methodological quality, LBP definition, and diabetes type. The association between DM and LBP remained consistent and statistically significant across the majority of the subgroup analyses (S3 text: Fig. 19).

### Sensitivity analysis

Multiple sensitivity analyses were conducted to examine the reliability of the primary pooled association, including leave-one-out protocol, methodological quality filtering, and an assessment of the influence of both large-scale data [56] and statistical outliers [41].

In brief, no single study disproportionately dictated the direction or significance of the results (S3 text: Figures 20 and 21).

The leave-one-out analysis demonstrated high stability of the overall findings; the pooled OR remained statistically significant (*p* < 0.001) regardless of the systematic omission of any individual study.

Exclusion of the largest cohort (*N* = 959,360) [56] resulted in an increase of the pooled effect size (OR 1.72; 95% CI: 1.38–2.15). Additionally, omission of the statistical outlier [41] reduced the *I*^2^ from 95.8% to 90.3%, while the association remained significant (OR 1.53; 95% CI: 1.28–1.83).

After excluding Poor-quality studies, the Good/Fair subgroup (*k* = 13) remained significant (OR 1.51; 95% CI: 1.25–1.82), with a reduction in heterogeneity (*I*^2^ = 90.7%).

Further restricting the analysis to only Good quality studies (*k* = 3), the pooled OR was 1.37 (95% CI: 0.98–1.93). Statistical significance was not maintained. However, inter-study heterogeneity was reduced to 56.8%, compared to 95.8% in the primary analysis.

### Meta-regression

Due to observed high heterogeneity, a random-effects meta-regression was performed. In univariate models, mean age was identified as a significant moderator (*k* = 11; *β* = 0.0375; *p* = 0.033), accounting for 33.8% of the observed heterogeneity. No significant moderation was observed for mean BMI (*k* = 7; *β* = 0.0299; *p* = 0.722; *R*^2^ = 0.00%) or sex distribution (*k* = 15; *β* = 0.0170; *p* = 0.211; *R*^2^ = 2.82%). In a multiple meta-regression model (*k* = 7), mean age (*p* < 0.001) and mean BMI (0.009) were significant predictors of the association between DM and LBP, accounting for 85.7% of the observed heterogeneity (S3 text: Table 6).

### Longitudinal relationship

#### Direction DM as a predictor for LBP

Meta-analysis of three longitudinal studies [36, 39, 53] examined the risk of developing LBP among individuals with DM in comparison to non-DM peers. The pooled relative risk (RR) was 1.22 (95% CI: 0.91–1.62; I^2^ = 68.2%; S3 text: Fig. 22).

Two studies provided sufficient data for pooled sex-specific subgroup analysis [36, 53]. Among women, the analysis pointed to that individuals with DM may have a higher risk for developing LBP compared to those without DM (RR 1.42; 95% CI: 0.88–2.32) but the result was not statistically significant. Men with DM however demonstrated a significantly lower risk of developing LBP compared to their non-diabetic peers (RR 0.83; 95% CI: 0.71–0.96; S3 text: Fig. 22).

#### Direction LBP as a predictor for DM

Two studies investigated the risk of developing DM among individuals with LBP in comparison to non-LBP peers [46, 53]. The pooled RR was 1.15 (95% CI: 1.02–1.29). Subgroup analysis revealed that the risk of developing DM was only significant in women (RR = 1.31; 95% CI: 1.11–1.56) with LBP in comparison to non-LBP peers. In men, the risk was however similar (RR = 1.00; 95% CI: 0.77–1.29; S3 text: Fig. 23).

### Narrative presentation of the case-control studies

A narrative synthesis of the two case-control studies included from the search strategy are presented in S3 text.

### Certainty of evidence

The certainty of evidence for all primary outcomes was rated as Very Low based on the GRADE framework (S3 text; Table 7). Certainty was initially set at a low level due to the observational nature of the study designs. Cross-sectional findings were downgraded for large statistical heterogeneity (*I*^2^ > 95%), evidence of publication bias, and indirectness resulting from non-standardized LBP definitions. Regarding the longitudinal cohort studies, the assessment was further impacted by imprecision, with the confidence intervals crossing the null value. The evidence remained at a very low certainty level due to the limitations of observational study designs and inconsistent LBP definitions in half of the included cohort studies.

## Discussion

### Main findings

This systematic review and meta-analysis of 26 studies (28 datasets), involving 1.3 million participants, pointed to a potential bidirectional association between DM and LBP, characterized by higher prevalence and odds for LBP in adult individuals with DM, compared with those without DM. Age was identified as a moderator in the analysis. Furthermore, the presence of LBP was modestly associated with developing DM, though this association was potentially stronger in women (31%).

### The cross-sectional analysis: magnitude and association

The cross-sectional analyses revealed a significant bidirectional burden between both conditions. LBP was 8.7% points more prevalent in the DM population (95% CI: 4.4–13), while DM prevalence was 7.4% points higher in individuals with LBP compared to their respective peers (95% CI: 3.8–11.0). Superior to prevalence gap analyses, the meta-analysis revealed that diabetic individuals have 66% higher odds of prevalent LBP than their non-diabetic peers (OR 1.66; 95% CI:1.34–2.06). This association remained statistically significant across most subgroup- and sensitivity analyses, including the leave-one-out protocol (S3 text).

However, the sensitivity analyses offer further insights to the understanding of the clinical association. For the prevalence of LBP among those with DM, the trim-and-fill analysis adjusted the gap from 8.7% to 2.36% (95% CI: −2.72–7.43), which failed to reach statistical significance. Similarly, the OR for the association was adjusted from 1.66 to 1.21 (95% CI: 0.93–1.59). This reduction likely occurred due to inflation by the publication of smaller studies reporting high effect associations [57]. While this suggests that the burden might be more modest than the raw estimates implied, it does not necessarily negate the relationship, rather indicates that the current literature may be skewed towards reporting high effect associations [15, 42, 44, 47, 50] (S3 text). It should also be pointed out that the trim-and-fill approach relies on the assumption that the asymmetry is caused only by publication bias, which may not be the case, and outliers may also substantially influence the findings. The adjusted odds ratios may therefore be too conservative.

A similar pattern was observed when the analysis was restricted to Good-quality studies (k = 3). The direction of the effect remained positive with an OR of 1.37. However, statistical significance was lost and is most probably explained by the small number of studies included in the sensitivity analysis.

A notable limitation is that data extraction from the largest included cohort, Park et al. (*N* = 959.360), was restricted to lumbar disc disorder. By omitting other prevalent spinal pathologies, our reported OR of 1.66 may be an understated estimate. It is possible that a more comprehensive inclusion of the full spectrum of LBP-related conditions would increase the observed effect size and reinforce the statistical power of the overall analysis.

To address a part of the observed heterogeneity (I2 > 95%), univariate meta-regression identified mean age as a significant moderator (*p* = 0.033; R^2^ = 33.8%). While BMI was not significant in isolation, it emerged as a significant predictor when combined with age (*p* = 0.009, R^2^ = 85.7%). One theory is the increased insulin resistance with increased BMI [58], and decrease insulin sensitivity with age, although influenced by factors such as adiposity [59]. However, these findings must be interpreted with caution. The multivariable model was restricted to those seven studies [15, 41, 43, 47, 50, 51, 53] that provided complete data, which increases the risk of overfitting due to few studies [25].

### The longitudinal evidence

The longitudinal cohorts demonstrated a non-significant 22% increased risk of LBP in diabetics in comparison to non-diabetic peers (RR 1.22; 95% CI: 0.91–1.62; *I*^2^ = 68.2%). In secondary analysis a pronounce sex-based difference was found, most evident in the largest included cohort [36] (*N* = 139,002) where an elevated risk was seen in women (RR = 1.68; 95% CI: 1.46–1.94) while the opposite was found in men (RR = 0.83; 95% CI: 0.71–0.97). The results must be interpreted with reservation, though they suggest that sex may act as a potential modifier in the relationship between diabetes and spinal health.

Conversely, the presence of LBP modestly increased the risk of developing DM by 15% in comparison to non-LBP peers (RR = 1.15; 95% CI: 1.02–1.29; *I*^2^ = 0%). In further analyses, the increased risk for DM was stronger in women with LBP (RR = 1.31; 95% CI: 1.11–1.56), rather than men (RR = 1.00; 95% CI: 0.77–1.29).

### Biological mechanisms

Our findings suggest that diabetes mellitus impacts low back pain. Even though we did not examine the mechanism it is interesting to note that hyperglycemia promotes the accumulation of advanced glycation end products which stiffen bone collagen and reduce its ability to absorb mechanical stress [60]. Simultaneously, elevated proinflammatory cytokines and oxidative stress accelerate disc degeneration and bone resorption [60]. Furthermore, the increased risk observed in women with DM may be attributed to more pronounced low-grade systematic inflammation [36, 61]. Female sex hormones, specifically associated with the menopausal shift, have been linked with amplified inflammatory response, which further may exacerbate the metabolic dysfunction which in turn may increase the risk of LBP development [53, 61].

As LBP remains the leading global cause of years lived with disability [5], a consequence is persistent physical immobility due to chronic pain [62]. This reduction in physical activity is a well-established risk factor for metabolic decline, whereas maintaining physical activity levels serves as a protective factor against the development of DM [63, 64]. Lower levels of physical activity in general have been reported among women compared to men [65] and the sex-based divergence in our results, specifically the increased risk of DM in women with LBP, may originate from these activity-level differences. Reduced physical activity due to LBP, may also accelerates the metabolic progression identified in women.

### Comparison with previous systematic reviews

Our study expands the existing evidence for the DM-LBP association. While Pozzobon et al. (2019) [53] reported an OR of 1.35 (*N* = 165,445), our analysis identified a higher point estimate of 1.66 (*N* = 1.3 million). Our larger and more geographically diverse sample likely provides a more comprehensive reflection of the global burden, even though our estimate may be influenced by the large heterogeneity and potential publication bias identified in the analyses. While previous review found temporal link inconclusive using a single longitudinal study, our expansion to four datasets allowed us to identify a potential bidirectional relationship.

Regarding prevalence of DM in LBP populations, our estimate of 20.2% is more conservative than the 27% reported by Deng et al. (2025) [66], likely due to our larger sample size. Notably, Deng identified male gender as a risk factor in their cross-sectional analysis, which contrasts with our findings. The results from our longitudinal findings, with the increased sample size, allowed us to identify sex as a potential modifier rather than a simple risk factor.

Beyond the scale, this study adds important information based on the geographic reach, detailed subgroup analyses, and the comparative identification of prevalence gaps between DM and LBP populations.

### Strengths and limitations

To our knowledge, this is the largest meta-analysis to date on the DM-LBP association, including over 1.3 million participants across four continents and 17 countries. The global scope and the identification of a potential bidirectional relationship, enhance the generalizability and provide a more dynamic understanding of the association of DM and LBP.

A primary limitation of this study is the *Very Low* certainty of evidence for all outcomes, largely driven by the statistical heterogeneity (*I*^2^ > 95%) and the observational nature of the included studies. To address this challenge, we utilized a meta-regression, which explained 85.7% of observed heterogeneity. Secondly, sensitivity analyses indicated a high risk of publication bias, suggesting that raw estimates may be inflated by smaller studies reporting high effect sizes. While our trim-and-fill analysis resulted in adjusted estimates that cross the null, these results may be viewed as a conservative lower bound of the association. The trim-and-fill approach assumes that funnel plot asymmetry is caused solely by publication bias, ignoring the impact of large heterogeneity, thus underestimating the relationship. Consequently, the actual global association may lie between our raw and adjusted estimates. Lastly, the non-standardized definitions of LBP across the studies introduce clinical indirectness to the results.

### Clinical significance and future direction

Our findings point to an integrated management of metabolic and musculoskeletal health, at least regarding LBP. Clinicians may implement metabolic screening for chronic LBP patients and spinal health monitoring for women with DM.

To facilitate future research, standardized clinical definitions for LBP should be used to reduce heterogeneity. There is also a need to distinguish between Type 1 and Type 2 DM, as current literature largely lacks specific data on Type 1 DM. Finally, investigating the pathobiological pathways behind the found association and the potential sex-based risk difference is essential to open opportunities for specific prevention and treatment strategies.

## Conclusion

This review points to a potential bidirectional association between diabetes and low back pain; however, given the low certainty of the underlying evidence, these results must be interpreted cautiously. Limited longitudinal data also suggest that LBP was modestly associated (15%) with higher risk of developing DM compared to non-LBP peers – potentially pointing to a stronger association in women (31%). These findings may be considered in the clinical setting for both patients with DM and for those with LBP. The low certainty of evidence highlights the critical need for more standardized, high-quality longitudinal research.

## Supplementary Information

Supplementary Material 1.

Supplementary Material 2.

Supplementary Material 3.

Supplementary Material 4.

Supplementary Material 5.

## Data Availability

The data generated or analyzed during this study are included in this published article and additional files.

## Abbreviations

AHRQ: Agency for Healthcare Research and Quality
BMI: Body Mass Index
CI: Confidence intervals
DM: Diabetes mellitus
GRADE: Grading of Recommendations Assessment, Development and Evaluation
HR: Hazard Ratio
ID: Identification
LBP: Low back pain
MeSH: Medical Subject Headings
NOS: Newcastle Ottawa Scale
OR: Odds ratio
PICO: Patient (/Population/Problem), Intervention, Comparison(/Control), Outcome(/Exposure)
PRISMA: Preferred Reporting Items for Systematic Reviews and Meta-analysis
PROSPERO: International Prospective Register of Systematic Reviews
RCT: Randomized controlled trials
RQ: Research question(s)
RR: Relative risk
S: Supplementary file (e.g., S1 Text, S2 Text, S3 Text)
SQR: Secondary research question(s)
YLD: Years lived with disability
WHO: World Health Organization
