# Supplementary material for: The association between diabetes mellitus and low back pain: a systematic review and meta-analysis
Source: BMC Musculoskelet Disord. 2026 Jul 14;27:611. doi: 10.1186/s12891-026-10226-z (PMC13377725; doi:10.1186/s12891-026-10226-z)
Supplement: Supplementary file 1 — Supplementary Material 1. [file 12891_2026_10226_MOESM1_ESM.pdf]

The Association Between Diabetes and Low Back Pain: A Study of Prevalence - A systematic review.

Muje El Noaimi, Björn Rosengren, Niyaz Hareni

To enable PROSPERO to focus on COVID-19 submissions, this registration record has undergone basic automated checks for eligibility and is published exactly as submitted. PROSPERO has never provided peer review, and usual checking by the PROSPERO team does not endorse content. Therefore, automatically published records should be treated as any other PROSPERO registration. Further detail is provided here.

Citation
Muje El Noaimi, Björn Rosengren, Niyaz Hareni. The Association Between Diabetes and Low Back Pain: A Study of Prevalence - A systematic review.. PROSPERO 2024 Available from https://www.crd.york.ac.uk/prospero/display_record.php?ID=CRD42025643242

PREVIEW

REVIEW TITLE AND BASIC DETAILS

Review title
The Association Between Diabetes and Low Back Pain: A Study of Prevalence - A systematic review.

Review objectives
I. Explore the bi-directional association between diabetes and low back pain.
RQ1: What is the occurrence of low back pain in diabetic individuals compared to non-diabetic individuals ?
RQ2: What is the prevalence of diabetes in individuals with low back pain?

Keywords
Low back pain; Degenerative spine disease; Diabetes; Prevalence

SEARCHING AND SCREENING

Searches
Sources: PubMed, CINAHL and EMBASE.
Search date: Inception to September 2024.
Restriction: English, Publication date 2000-present.
Manual search in reference lists of included articles and identified reviews. Grey literature search, sources: Google Scholar

Study design
Data from cohort studies, randomized controlled trials (RCT), case-control studies, twin and cross-sectional studies will be included in this review.

ELIGIBILITY CRITERIA

Condition or domain being studied
Diabetes is globally ranked the eighth leading cause of death and disability in the world. Low back pain is the leading cause of years lived with disability. Previous studies suggest that low back pain may be more common among people with diabetes. This literature review will summarize the prevalence of low back pain in individuals with diabetes in comparison to non-diabetic individuals and the prevalence of diabetes in individuals with low back pain.

Population

Included: 1) individuals ≥18 years of age having diabetes mellitus type 1 or type 2 (or similar forms of diabetes) and low back pain (or spinal diagnosis causing LBP); 2) prediabetic patients with low back pain (or spinal diagnosis causing LBP).
Excluded: i) individuals with gestational diabetes; ii) patients with other spine pathology such as fractures, cancerous, infectious and post-traumatic arthritis; iii) patients with rheumatoid arthritis;

Intervention(s) or exposure(s)

Prevalence (Occurrence) of low back pain in diabetic individuals compared to non-diabetic individuals.
Prevalence of diabetes in individuals with low back pain.

Comparator(s) or control(s)

Individuals with diabetes and low back pain will be compared to individuals without diabetes with low back pain.

OUTCOMES TO BE ANALYSED

Main outcomes

Prevalence/Occurence.

Additional outcomes

None.

DATA COLLECTION PROCESS

Data extraction (selection and coding)

The search criteria and strategy was produced with the help of an expert in systematic reviews (MB). The first author (ME) will screen according to title and abstract. If any doubt, the other authors will decide whether to include/exclude the study. Two authors (ME + NH) will read the full-text reviews and screen independently. Data will be extracted from the studies by a standardized extraction form created by the research team. Disagreements will be solved within the research team with the final decision made by BR. Covidence Software Program will be used for screening. Microsoft Word will be used for managing the data with calculation made through SPSS software.
Extracted data will include:
• Descriptive details – Author(s), year of publication, country
• Study design
• Study population size including the diabetic population.
• Demographics – gender, mean age or range, smoking, BMI, type of low back pain (if defined), duration of low back pain, type of diabetes (if defined), duration of diabetes, HbA1c.
• Outcome - prevalence/occurence data.

Risk of bias (quality) assessment

The studies will be assessed with the appropriate assessment tools according to the type of study.

PLANNED DATA SYNTHESIS

Strategy for data synthesis

The amount and quality of the collected data will determine appropriate analyses to answer the above mentioned research questions. Meta-analysis is planned to be conducted if possible and relevant, otherwise other quantitative analysis will be made.

Analysis of subgroups or subsets

Separate analysis will be made for both type prediabetes, type 1 and type 2 diabetes, if possible.

REVIEW AFFILIATION, FUNDING AND PEER REVIEW

Review team members

- Dr Muje El Noaimi, Clinical and Molecular Osteoporosis Research Unit, Departments of Orthopedics and Clinical Sciences, Lund University, Skåne University Hospital, Malmö and Departments of Orthopaedics, Halland Hospital
- Professor Björn Rosengren, Clinical and Molecular Osteoporosis Research Unit, Departments of Orthopedics and Clinical Sciences, Lund University, Skåne University Hospital, Malmö
- Dr Niyaz Hareni, Clinical and Molecular Osteoporosis Research Unit, Departments of Orthopedics and Clinical Sciences, Lund University, Skåne University Hospital, Malmö and Department of Orthopaedics, Halland Hospital

Review affiliation

Clinical and Molecular Osteoporosis Research Unit, Departments of Orthopedics and Clinical Sciences, Lund University, Skåne University Hospital, Malmö and Departments of Orthopaedics, Halland Hospital

Funding source

The supervisor (BR) supplies the doctoral student (ME) with a personal lap-top with relevant general and special software as well as access to expert resources regarding information and communication technology.

Named contact

Muje El Noaimi. Träslövsvägen 68, 432 37 Varberg
muje.el_noaimi@med.lu.se

TIMELINE OF THE REVIEW

Review timeline

Start date: 01 October 2023. End date: 01 April 2025

Date of first submission to PROSPERO

25 January 2025

Date of registration in PROSPERO

05 February 2025

AVAILABILITY OF FULL PROTOCOL

Availability of full protocol

No preview available

CURRENT REVIEW STAGE

Publication of review results

The intention is to publish the review once completed.The review will be publcished in English

Stage of the review at this submission

Review stage	Started	Completed
Pilot work	✓	✓
Formal searching/study identification	✓	✓
Screening search results against inclusion criteria	✓	✓
Data extraction or receipt of IPD	✓	✓
Risk of bias/quality assessment	✓	✓
Data synthesis	✓	

Review status

The review is currently planned or ongoing.

ADDITIONAL INFORMATION

Additional information

Different research questions although a similiar topic has been studied. An update of prevalence. A new research question that has not been studied before.

Collaborators

- **Miss Maria Björklund**, Library & ICT, Faculty of Medicine, Lund University

PROSPERO version history

- Version 1.1 published on 05 Feb 2025
- Version 1.0 published on 05 Feb 2025

Review conflict of interest

None known

Country

Sweden

Revision note

Updating the review such as data synthesis.

Disclaimer

The content of this record displays the information provided by the review team. PROSPERO does not peer review registration records or endorse their content.

PROSPERO accepts and posts the information provided in good faith; responsibility for record content rests with the review team. The owner of this record has affirmed that the information provided is truthful and that they understand that deliberate provision of inaccurate information may be construed as scientific misconduct.

PROSPERO does not accept any liability for the content provided in this record or for its use. Readers use the information provided in this record at their own risk.

Any enquiries about the record should be referred to the named review contact
