# Supplementary material for: The association between diabetes mellitus and low back pain: a systematic review and meta-analysis
Source: BMC Musculoskelet Disord. 2026 Jul 14;27:611. doi: 10.1186/s12891-026-10226-z (PMC13377725; doi:10.1186/s12891-026-10226-z)
Supplement: Supplementary file 2 — Supplementary Material 2. [file 12891_2026_10226_MOESM2_ESM.pdf]

Maria Björklund, Librarian / Information specialist
Library & ICT, Faculty of Medicine
Lund University
maria.bjorklund@med.lu.se

Lund University
Faculty of Medicine

SEARCH REPORT

PROJECT INFORMATION

REQUEST DATE	NAME	INSTITUTION/ORGANISATION
2024-01-15	Muje El Noaimi<br>Niyaz Hareni<br>Björn Rosengren	Orthopedic Surgery, Lund university and Skåne University Hospital

PROJECT TIME FRAME – DESIRED DATE OF DELIVERY

DESCRIBE RESEARCH QUESTION AND PURPOSE WITH THE SEARCH

Systematic review (no similar reviews registered in Prospero)

Diabetes and low back pain- associations
Outcomes after surgical or non-surgical therapy

STRUCTURED RESEARCH QUESTION

PROVIDE THE RESEARCH QUESTION IN A STURCTURED FORMAT
:PICO(S), PEO OR IN FREE ORDER

We aim to:

I. Explore the bi-directional association between diabetes and low back pain II.
II. Summarize the surgical and non-surgical outcomes of patients with diabetes and low back pain.

We defined several research questions (RQ):

RQ1: What is the prevalence of low back pain in diabetic patients compared to non-diabetic patients?

RQ2: What is the prevalence of diabetes in patients with low back pain?

RQ3: What is the outcome for diabetic patients with low back pain treated without surgery and how does this compare to non-diabetic patients?

RQ4: What is the outcome after surgery for diabetic patients with low back pain and how does this compare to non-diabetic patients?

REFERENCES TO KEY ARTICLES – EXAMPLES OF YOU WANT TO HAVE INCLUDED IN THE SEARCH RESULT (3-5 ARTICLES)

See project plan- included references
Two test sets for sensitivity test available- 10 highly relevant papers, and 410 eligible papers respectively.

DESIRED DELIVERY FORMAT (ENDNOTE, WORD, PDF, COVIDENCE)

EndNote library

Covidence- xml file for upload

SEARCH STRATEGIES

The search strategy documentation is to be cited as below, for protocol or publication in journal as appendix. The documentation is structured according to PRISMA 2020, international standard for systematic reviews. Please note that the information specialist delivers the parts required for search strategy documentation, additional method information may also be required.

See PRISMA 2020 for further information:
http://prisma-statement.org/
http://prisma-statement.org/Extensions/Searching

General annotation search strategies:
Two search strategies are developed to capture RQ 1+2, and RQ3+4 respectively.

Sensitivity tests were performed- papers in project plan cross-checked with search strategies.

Databases

PubMed (National Library of Medicine)

Date of search: 2024, February 5

[Diabetes terms]

#1 (("diabetes mellitus"[MeSH Terms]) OR ("diabetes mellitus, type 1"[MeSH Terms])) OR ("diabetes mellitus, type 2"[MeSH Terms]) OR (Glucose Intolerance[MeSH Terms]) OR (Prediabetic State[MeSH Terms]) OR (Diabetes Complications[MeSH Terms]) OR (Insulin Resistance[MeSH Terms])

592174 records

#2 Diabetes mellitus[Title/Abstract] OR diabet*[Title/Abstract] OR type 1 diabetes[Title/Abstract] OR type 2 diabetes[Title/Abstract] OR T1D[Title/Abstract] OR T2D[Title/Abstract] OR T1DM[Title/Abstract] OR

T2DM[Title/Abstract] OR insulin dependent[Title/Abstract] OR non insulin dependent[Title/Abstract] OR non-insulin dependent[Title/Abstract] OR glucose intolerance[Title/Abstract] OR prediabet*[Title/Abstract] OR diabetes complication*[Title/Abstract] OR insulin resistance[Title/Abstract]
862979 records

#3 #1 OR #2
948422 records

[Low back pain terms]
#4 ((((("low back pain"[MeSH Terms]) OR ("intervertebral disc degeneration"[MeSH Terms])) OR ("intervertebral disc displacement"[MeSH Terms])) OR ("hernia"[MeSH Terms])) OR ("spinal stenosis"[MeSH Terms]) OR ("spondylolisthesis"[MeSH Terms])) OR (Spondylosis[MeSH Terms]) OR ("sciatica"[MeSH Terms]) OR musculoskeletal pain[MeSH Terms]
138131 records

#5 Low back pain[Title/Abstract] OR back pain[Title/Abstract] OR low back ache[Title/Abstract] OR LBP[Title/Abstract] OR (degenerat*[Title/Abstract] AND (disc[Title/Abstract] OR disk[Title/Abstract])) OR degenerative spine*[Title/Abstract] OR disc hernia*[Title/Abstract] OR spinal stenos*[Title/Abstract] OR Spin* Osteoarthritis[Title/Abstract] OR spondylolisthes*[Title/Abstract] OR spondylosis[Title/Abstract] OR lumbar[Title/Abstract] OR lumbago[Title/Abstract] OR sciatic*[Title/Abstract] OR invertebra* disc*[Title/Abstract] OR musculoskeletal pain[Title/Abstract]
244712 records

#6 #4 OR #5
326545 records

[Prevalence- correlation terms]
#7 ("prevalence"[MeSH Terms]) OR ("incidence"[MeSH Terms])
625843 records

#8 Prevalence[Title/Abstract] OR incidence[Title/Abstract] OR Correlat*[Title/Abstract] OR associat*[Title/Abstract] OR relat*[Title/Abstract] OR causal[Title/Abstract] OR subgroup*[Title/Abstract] OR risk analysis[Title/Abstract] OR onset[Title/Abstract]
12580305 records

#9 #7 OR #8
12670541 records

[Surgery terms]
#10 (((((("general surgery"[MeSH Terms]) OR ("surgery"[MeSH Subheading]))) OR ("low back pain/surgery"[MeSH Terms])) OR ("spinal diseases/surgery"[MeSH Terms])) OR ("spinal injuries/surgery"[MeSH Terms])) OR ("spinal stenosis/surgery"[MeSH Terms])
2401340 records

#11 Surgery[Title/Abstract] OR surgical[Title/Abstract] OR surg*[Title/Abstract]
2448900 records

#12 #10 OR #11
3697948 records

[Combination search Diabetes AND Low Back Pain AND Prevalence-correlation]
#13 #3 AND #6 AND #9
4634 records

#14 #13 NOT animal*
3606 records

#15 #14 NOT gestational
3481 records

Filter: Publication date 2000- present (date of search)
3199 records

Filter: English
3034 records

[Combination search Diabetes AND Low Back Pain AND Surgery]
#16 #3 AND #6 AND #12
1900 records

#17 #15 NOT animal*
1792 records

#18 #17 NOT gestational
1767 records

Filter: Publication date 2000- present (date of search)
1560 records

Filter: English
1466 records

Annotation PubMed search:
Overlap (duplicates) between searches #15 + #18 (1046 records), will be removed in Covidence.
In total, 3454 unique records are retrieved in the PubMed searches with selected filters.

Embase.com (Elsevier, 1947-present)
Date of search: 2024 February 5

[Diabetes terms]
'diabetes mellitus'/exp OR 'insulin dependent diabetes mellitus'/exp OR 'non insulin dependent diabetes mellitus'/exp OR 'glucose intolerance'/exp OR 'impaired glucose tolerance'/exp OR 'diabetic complication'/exp OR 'insulin resistance'/exp
1393393 records

#2 'diabetes mellitus':ab,ti OR diabet*:ab,ti OR 'type 1 diabetes':ab,ti OR 'type 2 diabetes':ab,ti OR t1d:ab,ti OR t2d:ab,ti OR t1dm:ab,ti OR t2dm:ab,ti OR 'insulin dependent':ab,ti OR 'non insulin dependent':ab,ti OR 'non-insulin dependent':ab,ti OR 'glucose intolerance':ab,ti OR prediabet*:ab,ti OR 'diabetes complication*':ab,ti OR 'insulin resistance':ab,ti
1309188 records

#3 #1 OR #2
1610807 records

[Low back pain terms]
#4 'low back pain'/exp OR 'intervertebral disk degeneration'/exp OR 'hernia'/exp OR 'vertebral canal stenosis'/exp OR 'spondylosis'/exp OR 'spondylolisthesis'/exp OR 'sciatica'/exp OR 'musculoskeletal pain'/exp
371235 records

#5 'low back pain':ab,ti OR 'back pain':ab,ti OR 'low back ache':ab,ti OR lbp:ab,ti OR (degenerat*:ab,ti AND (disc:ab,ti OR disk:ab,ti)) OR 'degenerative spine*':ab,ti OR 'disc hernia*':ab,ti OR 'spinal stenos*':ab,ti OR 'spin* osteoarthritis':ab,ti OR spondylolisthes*:ab,ti OR spondylosis:ab,ti OR lumbar:ab,ti OR lumbago:ab,ti OR sciatic*:ab,ti OR 'invertebra* disc*':ab,ti OR 'musculoskeletal pain':ab,ti
319022 records

#6 #4 OR #5
559817 records

[Prevalence-incidence terms]
#6 'prevalence'/exp OR 'incidence'/exp
1637694 records

#7 prevalence:ab,ti OR incidence:ab,ti OR correlat*:ab,ti OR associat*:ab,ti OR relat*:ab,ti OR causal:ab,ti OR subgroup*:ab,ti OR 'risk analysis':ab,ti OR onset:ab,ti
16315694 records

#8 #6 OR #7
16562731 records

[Surgery terms]
#9 'surgery'/exp
6297775 records

#10 'surgery'/lnk
2589765 records

#11 surgery:ab,ti OR surgical:ab,ti OR surg*:ab,ti
3304870 records

#12 #9 OR #10 OR #11
7493919 records

[Combination search Diabetes AND Low Back Pain AND Prevalence-correlation]

#13 #3 AND #6 AND #8
14823 records
#14 #13 NOT animal*
13309 records

#15 #14 NOT gestational
13029 records

#16 #15 AND [embase]/lim NOT ([embase]/lim AND [medline]/lim)
6404 records

NOT 'conference abstract':it
2326 records

Filter: Publication date 2000- present
2284 records

Filter: English
2118 records

[Combination search Diabetes AND Low Back Pain AND Surgery]
#17 **#3 AND #6 AND #12**
10603 records

#18 #17 NOT animal*
10224 records

#19 #18 NOT gestational
10037 records

AND [embase]/lim NOT ([embase]/lim AND [medline]/lim)
5222 records

NOT 'conference abstract':it
2117 records

Filter: Publication date 2000- present
2082 records

Filter: English
1967 records

Annotation Embase search:
Duplicates from Medline removed in search, Covidence may detect additional duplicates.
Overlap between search #16 + #19 is 1014 records**.** In total 3071 unique records in Embase.

CINAHL Complete (EbscoHost, inception to present)

Date of search: 2024, February 5

[Diabetes terms]
#1 (MH "Diabetes Mellitus+") OR (MH "Diabetes Mellitus, Type 1") OR (MH "Diabetes Mellitus, Type 2") OR (MH "Glucose Intolerance") OR (MH "Insulin Resistance+") OR (MH "Prediabetic State")
212845 records

#2 TI (Diabetes mellitus OR diabet* OR type 1 diabetes OR type 2 diabetes OR T1D OR T2D OR T1DM OR T2DM OR insulin dependent OR non insulin dependent OR non-insulin dependent OR glucose intolerance OR prediabet* OR diabetes complication* OR insulin resistance) OR AB (Diabetes mellitus OR diabet* OR type 1 diabetes OR type 2 diabetes OR T1D OR T2D OR T1DM OR T2DM OR insulin dependent OR non insulin dependent OR non-insulin dependent OR glucose intolerance OR prediabet* OR diabetes complication* OR insulin resistance)
262629 records

#3 #1 OR #2
309079 records

[Low back pain terms]
#4 (MH "Low Back Pain") OR (MH "Intervertebral Disk Displacement") OR (MH "Hernia+") OR (MH "Spinal Stenosis") OR (MH "Spondylolisthesis") OR (MH "Spondylosis+") OR (MH "Sciatica") OR (MH "Musculoskeletal Pain")
44396 records

#5 TI (Low back pain OR back pain OR low back ache OR LBP OR (degenerat* AND (disc OR disk)) OR degenerative spine* OR disc hernia* OR spinal stenos* OR Spin* Osteoarthritis OR spondylolisthes* OR spondylosis OR lumbar OR lumbago OR sciatic* OR invertebra* disc* OR musculoskeletal pain) OR AB (Low back pain OR back pain OR low back ache OR LBP OR (degenerat* AND (disc OR disk)) OR degenerative spine* OR disc hernia* OR spinal stenos* OR Spin* Osteoarthritis OR spondylolisthes* OR spondylosis OR lumbar OR lumbago OR sciatic* OR invertebra* disc* OR musculoskeletal pain)
84838 records

#6 #4 OR #5
102506 records

[Prevalence-incidence terms]
#7 (MH "Prevalence") OR (MH "Incidence")
183390 records

#8 TI (Prevalence OR incidence OR Correlat* OR associat* OR relat* OR causal OR subgroup* OR risk analysis OR onset) OR AB (Prevalence OR incidence OR Correlat* OR associat* OR relat* OR causal OR subgroup* OR risk analysis OR onset)
2626742 records

#9 #7 OR #8
2663380 records

[Surgery terms]

#10 (MH "Surgery, Operative+")
773943 records

#11 TI (Surgery OR surgical OR surg*) OR AB (Surgery OR surgical OR surg*)
509752 records

#12 #10 OR #11
1022495 records

[Combination search Diabetes AND Low Back Pain AND Prevalence-correlation]
#13 #3 AND #6 AND #9
1401 records

#14 #13 NOT animal*
1257 records

#15 #14 NOT gestational
1240 records

Filters: Publication date 2000 to present, Academic journals, English
1164 records

[Combination search Diabetes AND Low Back Pain AND Surgery]
#16 #3 AND #6 AND #12
622 records

#17 #16 NOT animal*
579 records

#18 #17 NOT gestational
575 records

Filters: Publication date to present, Academic journals, English
537 records

Annotation CINAHL search:
Default is All fields if field/heading is not specified.
Overlap in CINAHL of 371, searhces retrieve 1330 unique records in CINAHL (#15 OR #18)

Total number of unique records from databases in EndNote before deduplication:
10296 records

General annotations:
Duplicates: 3464 duplicates indicated by EndNote, in total 6832 records for screening, Covidence may detect additional duplicates.
Sensitivity test of search strategy: Two test sets of 10 key highly relevant papers and 410 other relevant papers respectively were used, all 10 key papers were retrieved by search strategy. EndNote indicated 407 of 410 as duplicates, where 2 were found to be additional duplicates, the remaining paper was a background paper (Global burden of disease).
