# Supplementary material for: The association between diabetes mellitus and low back pain: a systematic review and meta-analysis
Source: BMC Musculoskelet Disord. 2026 Jul 14;27:611. doi: 10.1186/s12891-026-10226-z (PMC13377725; doi:10.1186/s12891-026-10226-z)
Supplement: Supplementary file 3 — Supplementary Material 3. [file 12891_2026_10226_MOESM3_ESM.pdf]

Table of content

PICO framework for Study Eligibility **2**

Newcastle-Ottawa scale and AHRQ standards **3**

The mapping of stars *4*

Risk of Bias assessment according to Newcastle Ottawa Scale and AHRQ with mapping thresholds and conversion to traffic-light plot. *5*

Continent demographics **7**

Overview of studies regarding country of origin and population based/non-population based **8**

BMI and Age distribution **9**

Prevalence of LBP in DM subgroup and non-DM subgroup **11**

Comparative analysis of LBP prevalence in DM vs non-DM and publication bias *12*

Prevalence of DM in LBP subgroup and non-LBP subgroup **13**

Comparative analysis of DM prevalence in LBP vs non LBP and publication bias *14*

The association between DM and LBP **15**

Publication bias *15*

Subgroup analysis *15*

Sensitivity analysis *16*

Meta regression *17*

Case-Control narrative presentation **17**

Longitudinal association **19**

The certainty of evidence **20**

PICO framework for Study Eligibility

**Component**	Description
**Population (P)**	Adults (≥ 18 years) with or without Diabetes Mellitus (DM) and/or Low Back Pain (LBP). LBP includes patients with various degenerative spinal conditions (e.g., spinal stenosis, disc herniation, spondylolisthesis) and all types of DM (Type 1, Type 2, and prediabetes).
**Exposure (E/I)**	The presence or clinical diagnosis of Diabetes Mellitus (or its subtypes) and/or the presence or diagnosis of Low Back Pain (or related degenerative spinal conditions).
**Comparison (C)**	Individuals without the condition of interest (e.g., non-diabetic individuals compared to diabetic individuals, or individuals without LBP compared to those with LBP).
**Outcomes (O)**	1. The prevalence of low back pain in diabetic versus non-diabetic populations.<br><br>2. The prevalence of diabetes among patients with low back pain versus without low back pain.<br><br>3. The incidence of low back pain in diabetic versus non-diabetic populations.<br><br>4. The incidence of diabetes in people with low back pain versus people without low back pain.
**Study Design (S)**	Observational studies, including cross-sectional, cohort, and case-control studies.
Note: *This PICO framework and the final analysis focus on the primary research questions regarding association and prevalence (RQ1 and RQ2). As specified in the Protocol Deviations section of the manuscript, the surgical and non-surgical treatment outcomes (RQ3 and RQ4) were ultimately excluded from the final synthesis.*	

Table 2 *– The definition of PICO framework (Population, Exposure, Comparison, Outcome and Study Design)*

Newcastle-Ottawa scale and AHRQ standards

The following are accepted thresholds for converting the Newcastle-Ottawa scales to AHRQ standards (Good, Fair, and Poor-quality studies):

Study Quality (cohort study, case-control study)

- **Good quality:** 3 or 4 stars in selection domain AND 1 or 2 stars in compatibility domain AND 2 or 3 stars in outcome domain.

- **Fair quality:** 2 stars in selection domain AND 1 or 2 stars in comparability domain AND 2 or 3 stars in outcome domain.

- **Poor quality:** 0 or 1 star in selection domain OR 0 stars in comparability domain OR 0 or 1 stars in outcome domain.

Study Quality (cross-sectional study)

- **Good quality:** 4 or 5 stars in selection domain AND 2 or 3stars in compatibility domain AND 2 or 3 stars in outcome domain.

- **Fair quality:** 2 or 3 stars in selection domain AND 2 or 3 stars in comparability domain AND 2 or 3 stars in outcome domain.

- **Poor quality:** 0 or 1 star in selection domain OR 0-1 stars in comparability domain OR 0 -1 stars in outcome domain.

The mapping of stars

The mapping of stars to the categories **Low, Some concerns,** and **High** (AHRQ standards).

Cohort/Case-Control Studies

- Selection Domain (Max 4 Stars)

3–4 Stars: Low Risk (Good)

2 Stars: Some Concerns (Fair)

0–1 Stars: High Risk (Poor)

- Comparability Domain (Max 2 Stars)

1-2 Stars: Low Risk (Good)

0 Stars: High Risk (Poor)

- Outcome/Exposure Domain (Max 3 Stars)

2–3 Stars: Low Risk (Good)

0–1 Stars: High Risk (Poor)

Cross-Sectional Studies (CS):

- Selection:

4–5 Stars: Low Risk (Good)

2–3 Stars: Some Concerns (Fair)

0–1 Stars: High Risk (Poor)

- Comparability:

2–3 Stars: Low Risk (Good)

0-1 Stars: High Risk (Poor)

- Exposure/Outcome:

2–3 Stars: Low Risk (Good)

0–1 Stars: High Risk (Poor)

Risk of Bias assessment according to Newcastle Ottawa Scale and AHRQ with mapping thresholds and conversion to traffic-light plot.

Study (design)	Selection	Comparability	Exposure/ Outcome	Total stars	Scale/Form
Alsubaie et al., 2024 (CS)	*	*	**	4/10	NOS Cross-sectional
Chang et al., 2022 (CS)	***	**	***	8/10	NOS Cross-sectional
Ha et al., 2014 (CS)	***	**	***	8/10	NOS Cross-sectional
Anekstein et al., 2010 (CS)	***	*	***	7/10	NOS Cross-sectional
Hassoon et al., 2017 (CS)	***	**	**	7/10	NOS Cross-sectional
Karacif et al., 2022 (CS)	**	**	*	5/10	NOS Cross-sectional
Ibrahim et al., 2022 (CS)	**	**	*	5/10	NOS Cross-sectional
Molsted et al., 2012 (CS)	****	*	*	6/10	NOS Cross-sectional
Olaosebikan et al., 2019 (CS)	****	*	***	8/10	NOS Cross-sectional
Abaraogu et al., 2017 (CS)	****	**	**	8/10	NOS Cross-sectional
Asadian et al., 2016 (CS)	***	*	**	6/10	NOS Cross-sectional
de Luca et al., 2023 (CS)	**	*	**	5/10	NOS Cross-sectional
Eivazi et al., 2012 (CS)	***	**	*	6/10	NOS Cross-sectional
Heuch et al., 2018 (CS)	***	**	**	7/10	NOS Cross-sectional
Iizuka et al., 2017 (CS)	**	**	**	6/10	NOS Cross-sectional
Al-Rudaini et al., 2022 (CS)	*	*	*	3/10	NOS Cross-sectional
Jena et al., 2022 (CS)	**	**	***	7/10	NOS Cross-sectional
Maeda et al., 2018 (CS)	***	**	***	8/10	NOS Cross-sectional
Real et al., 2019 (CS)	***	**	**	7/10	NOS Cross-sectional
Dario et al., 2017 (CS)	***	**	**	7/10	NOS Cross-sectional
Uesugi et al., 2013 (CS)	**	*	**	5/10	NOS Cross-sectional
Park et al., 2021 (CS)	****	*	***	9/10	NOS Cross-sectional
Shemesh et al., 2023 (CC)	***	**	***	8/9	NOS Case-control
Abbas et al., 2013 (CC)	***	**	**	7/9	NOS Case-control
Jacob et al., 2021 (RC)	****	*	**	7/9	NOS Cohort
Wang et al., 2021 (RC)	***	*	***	7/9	NOS Cohort
Heuch et al., 2018 (PC)	***	**	**	7/9	NOS Cohort
Dario et al., 2017 (PC)	****	**	*	7/9	NOS Cohort

Table 3 *- Risk of Bias assessment according to Newcastle Ottawa Scale.*
CS = Cross-sectional study. CC – Case-Control study. PC – Prospective cohort study. RC – Retrospective cohort study.

Study (Design)	Selection	Comparability	Exposure /Outcome	Quality	Overall according to trafficplot
Alsubaie et al., 2024 (CS)	High	Low	Low	Poor	High
Chang et al., 2022 (CS)	Some concerns	Low	Low	Fair	Some concerns
Ha et al., 2014 (CS)	Some concerns	Low	Low	Fair	Some concerns
Anekstein et al., 2010 (CS)	Some concerns	Low	Low	Fair	Some concerns
Hassoon et al., 2017 (CS)	Some concerns	Low	Low	Fair	Some concerns
Karacif et al., 2022 (CS)	Some concerns	Low	High	Poor	High
Ibrahim et al., 2022 (CS)	Some concerns	Low	High	Poor	High
Molsted et al., 2012 (CS)	Low	Low	High	Poor	High
Olaosebikan et al., 2019 (CS)	Low	Low	Low	Good	Low
Abaraogu et al., 2017 (CS)	Low	Low	Low	Good	Low
Asadian et al., 2016 (CS)	Some concerns	Low	Low	Fair	Some concerns
de Luca et al., 2023 (CS)	Some concerns	Low	Low	Fair	Some concerns
Eivazi et al., 2012 (CS)	Some concerns	Low	High	Poor	High
Heuch et al., 2018 (CS)	Some concerns	Low	Low	Fair	Some concerns
Iizuka et al., 2017 (CS)	Some concerns	Low	Low	Fair	Some concerns
Al-Rudaini et al., 2022 (CS)	High	Low	High	Poor	High
Jena et al., 2022 (CS)	Some concerns	Low	Low	Fair	Some concerns
Maeda et al., 2018 (CS)	Some concerns	Low	Low	Fair	Some concerns
Real et al., 2019 (CS)	Some concerns	Low	Low	Fair	Some concerns
Dario et al., 2017 (CS)	Some concerns	Low	Low	Fair	Some concerns
Uesugi et al., 2013 (CS)	Some concerns	Low	Low	Fair	Some concerns
Park et al., 2021 (CS)	Low	Low	Low	Good	Low
Shemesh et al., 2023 (CC)	Low	Low	Low	Good	Low
Abbas et al., 2013 (CC)	Low	Low	Low	Good	Low
Jacob et al., 2021 (RC)	Low	Low	Low	Good	Low
Wang et al., 2021 (RC)	Low	Low	Low	Good	Low
Heuch et al., 2018 (PC)	Low	Low	Low	Good	Low
Dario et al., 2017 (PC)	Low	Low	High	Poor	High

Table 4 - *AHRQ mapping and conversion to traffic-light colors*
CS = Cross-sectional study. CC – Case-Control study. PC – Prospective cohort study. RC – Retrospective cohort study.

Continent demographics

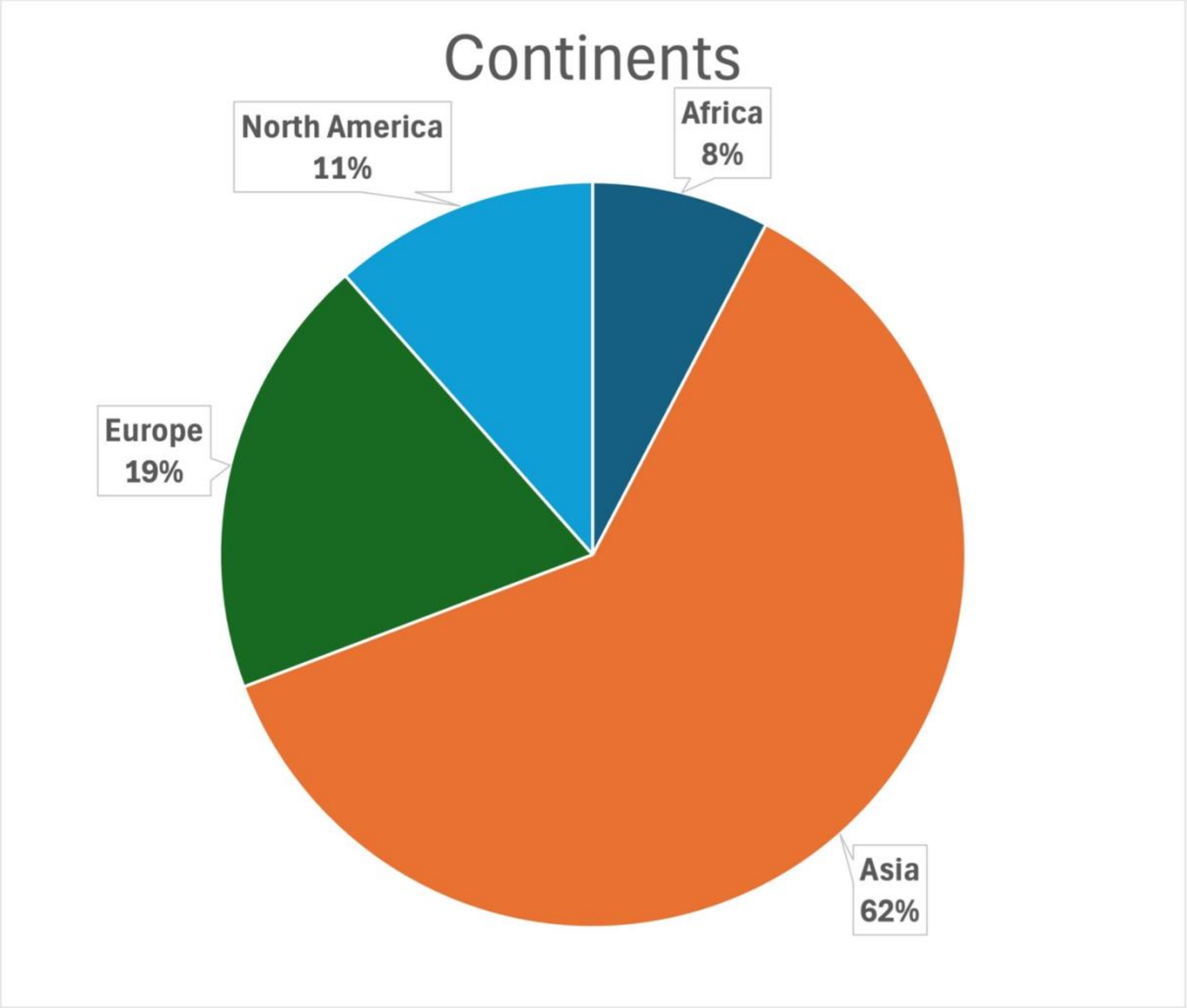

Figure 4 *- Geographical distribution of included studies by continent.*

Overview of studies regarding country of origin and population based/non-population based

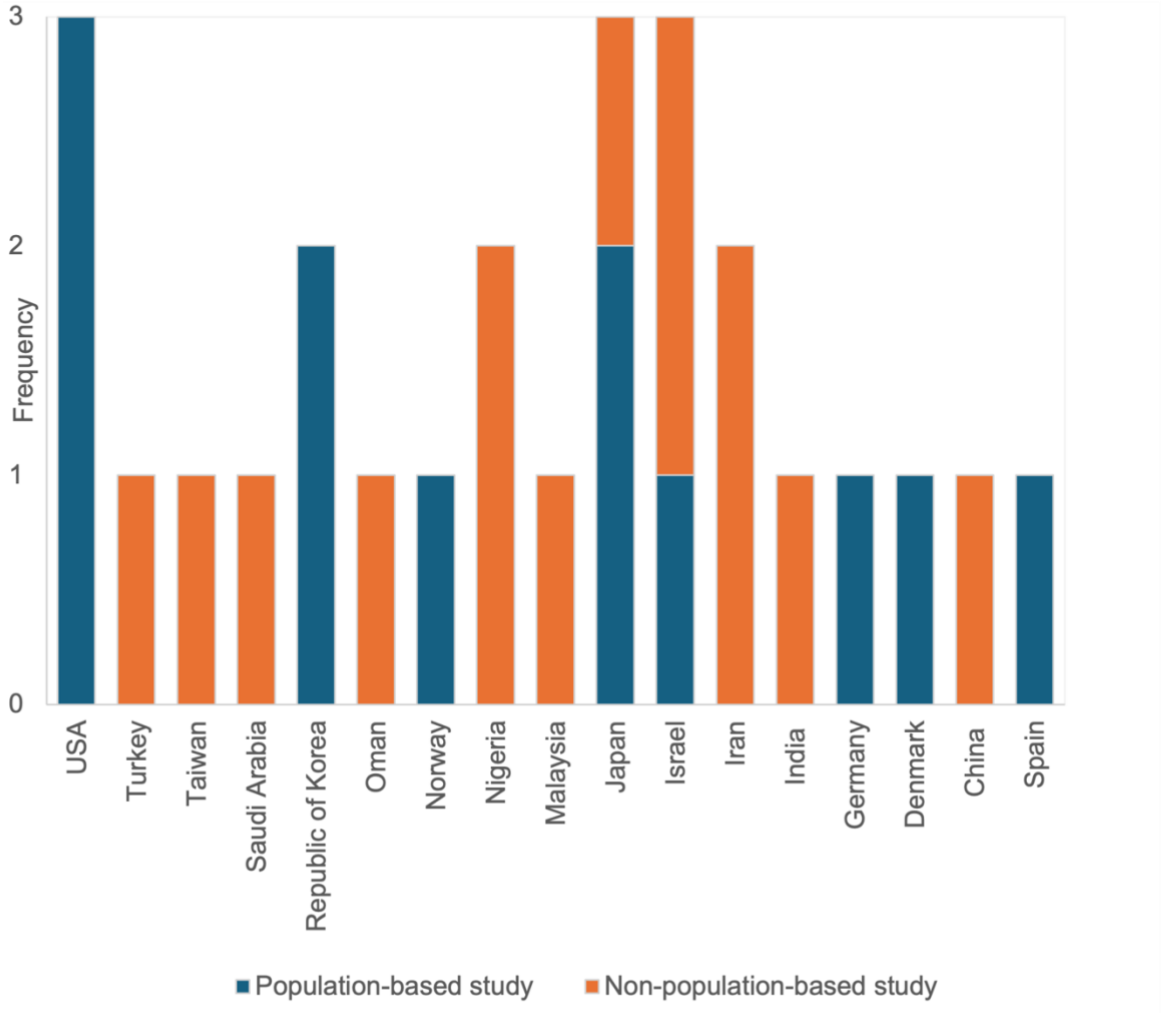

Figure 5 - *Study frequency per country, grouped after population-based or non-population-based studies*

BMI and Age distribution

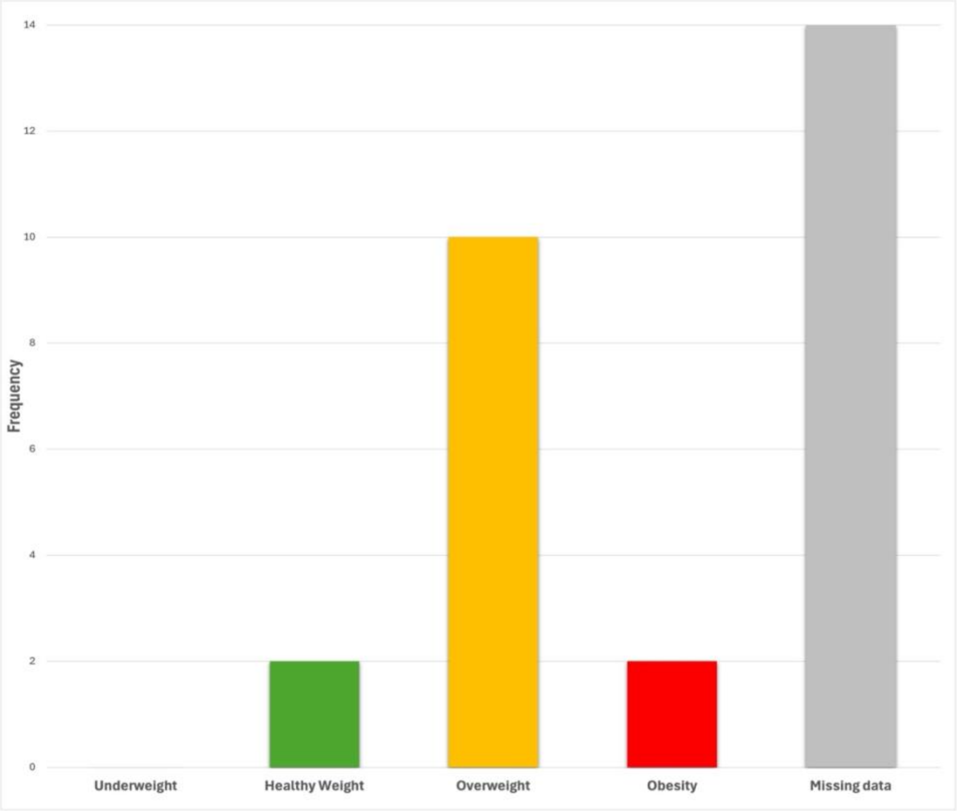

Figure 6 *– BMI distribution intervals according to data from the studies. Adult BMI categories [Internet]. CDC. 2024. Available from: https://www.cdc.gov/bmi/adult calculator/bmi-categories.html*

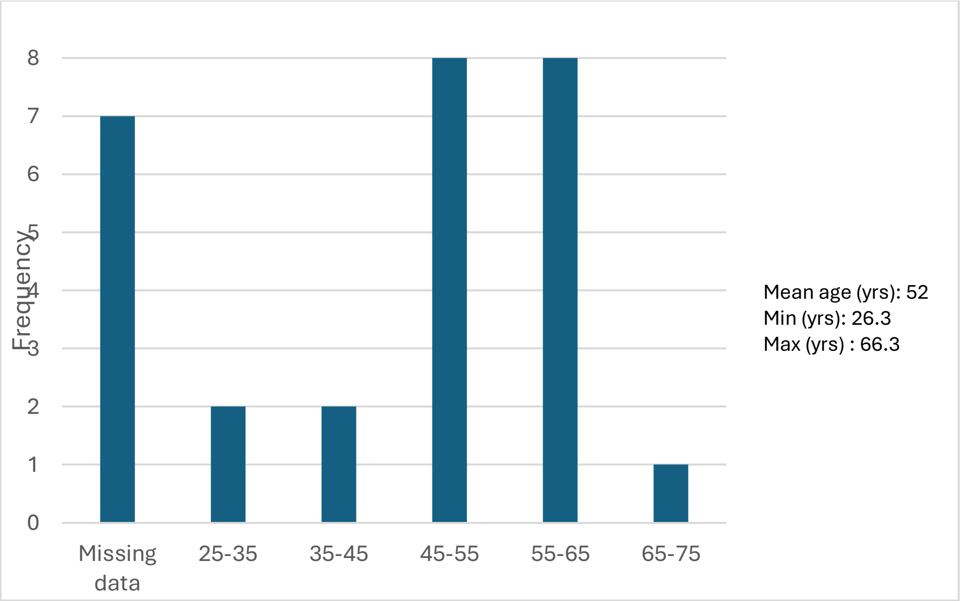

Figure 7 *– Age distribution intervals according to data from the studies.*
Note: Data from the two studies providing both cross-sectional and cohort data is included in the summarization of BMI and Age (Dario et al. (2017) and Heuch et al. (2018)).

Methodological quality – summary plot

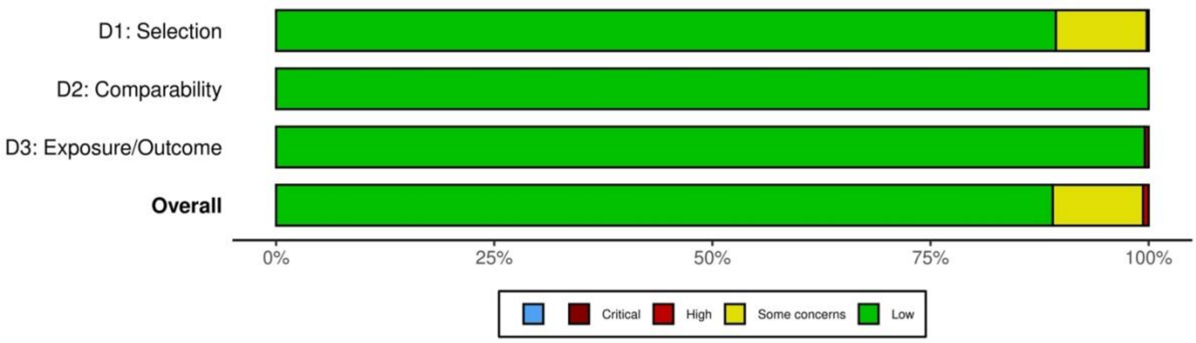

Figure 8 *– Summary plot for the cross-sectional studies*

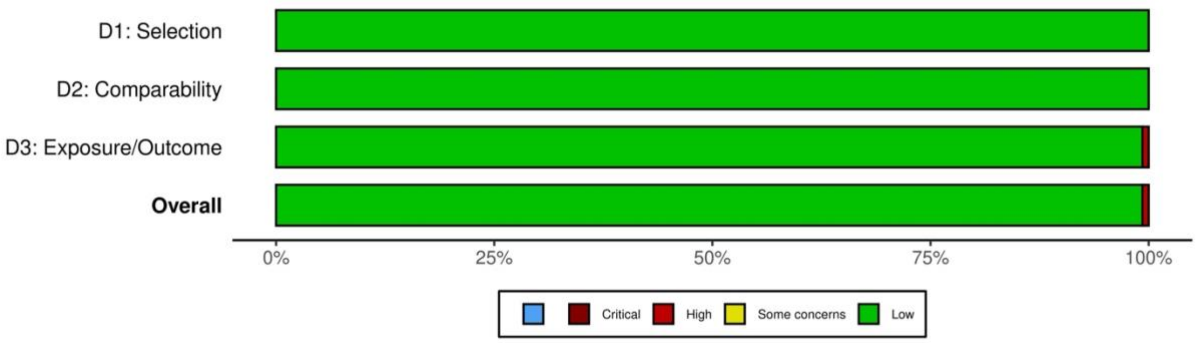

Figure 9 *– Summary plot for the cohort and case-control studies combined*

Prevalence of LBP in DM subgroup and non-DM subgroup

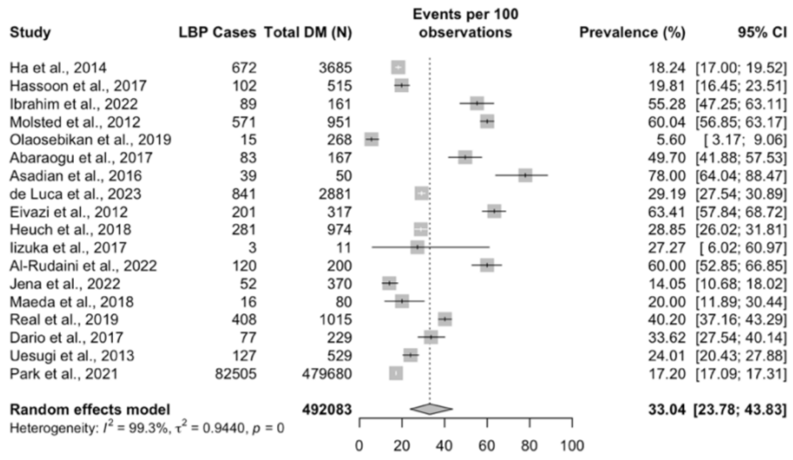

Figure 10 *– prevalence estimation with 95% CI in the DM population with LBP.*

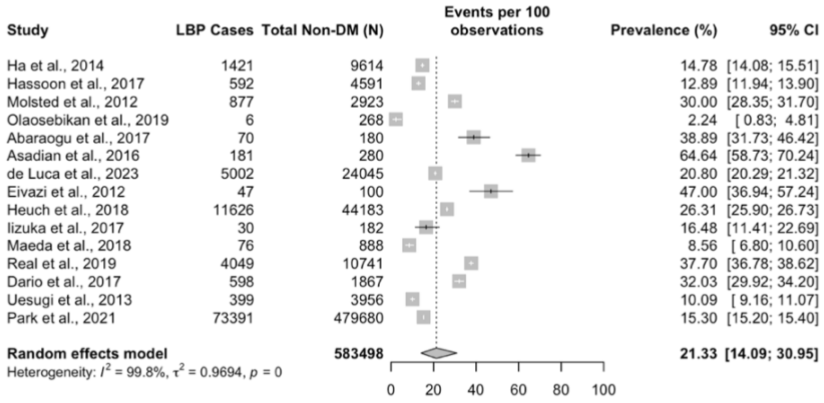

Figure 11 *– prevalence calculations with 95% CI in the non-DM population with LBP.*

Comparative analysis of LBP prevalence in DM vs non-DM and publication bias

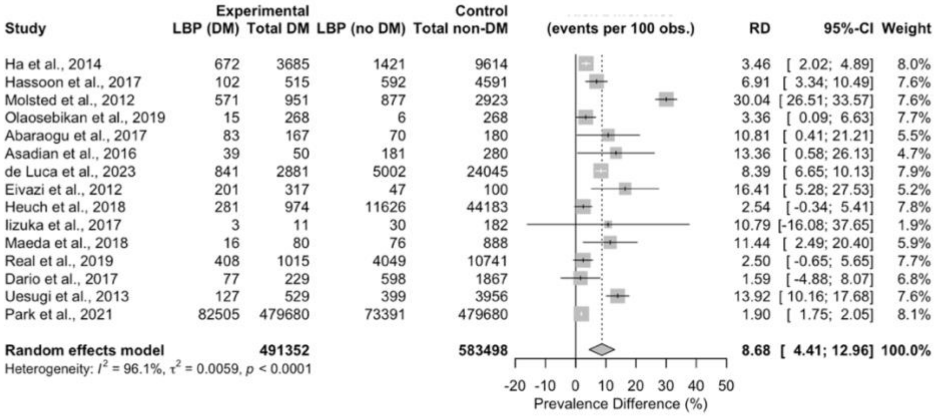

Study	Experimental LBP (DM)	Experimental Total DM	Control LBP (no DM)	Control Total non-DM	RD	95%-CI	Weight
Ha et al., 2014	672	3685	1421	9614	3.46	[2.02; 4.89]	8.0%
Hassoon et al., 2017	102	515	592	4591	6.91	[3.34; 10.49]	7.6%
Molsted et al., 2012	571	951	877	2923	30.04	[26.51; 33.57]	7.6%
Olaosebikan et al., 2019	15	268	6	268	3.36	[0.09; 6.63]	7.7%
Abaraogu et al., 2017	83	167	70	180	10.81	[0.41; 21.21]	5.5%
Asadian et al., 2016	39	50	181	280	13.36	[0.58; 26.13]	4.7%
de Luca et al., 2023	841	2881	5002	24045	8.39	[6.65; 10.13]	7.9%
Eivazi et al., 2012	201	317	47	100	16.41	[5.28; 27.53]	5.2%
Heuch et al., 2018	281	974	11626	44183	2.54	[-0.34; 5.41]	7.8%
Iizuka et al., 2017	3	11	30	182	10.79	[-16.08; 37.65]	1.9%
Maeda et al., 2018	16	80	76	888	11.44	[2.49; 20.40]	5.9%
Real et al., 2019	408	1015	4049	10741	2.50	[-0.65; 5.65]	7.7%
Dario et al., 2017	77	229	598	1867	1.59	[-4.88; 8.07]	6.8%
Uesugi et al., 2013	127	529	399	3956	13.92	[10.16; 17.68]	7.6%
Park et al., 2021	82505	479680	73391	479680	1.90	[1.75; 2.05]	8.1%
**Random effects model**		**491352**		**583498**	**8.68**	**[4.41; 12.96]**	**100.0%**

Heterogeneity: $I^2 = 96.1\%$, $\tau^2 = 0.0059$, $p < 0.0001$

Figure 12 – *prevalence difference for LBP in the DM vs non-DM groups (with 95% CI)*

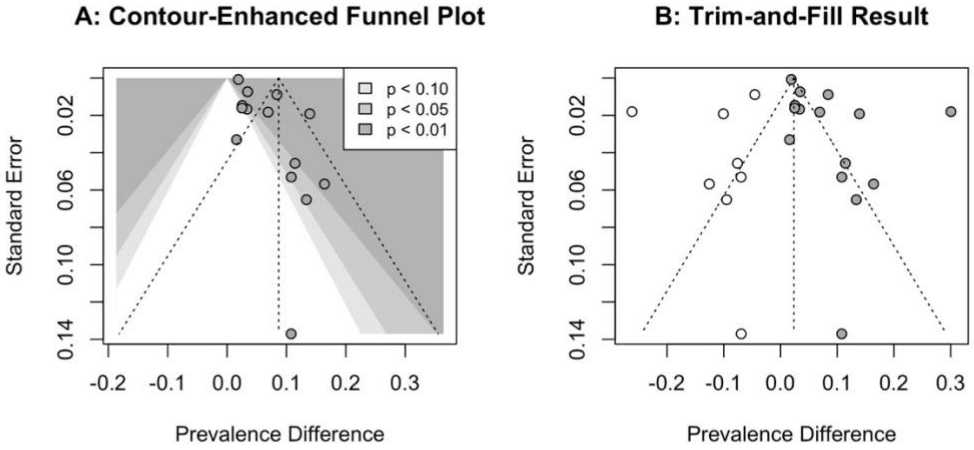

Figure 13 *- Publication bias. Assessment of Small-Study Effects: (A) The contour-enhanced funnel plot suggests that asymmetry is largely driven by studies in the significant zones. (B) A trim-and-fill sensitivity analysis was conducted to address the observed asymmetry, which simulated eight hypothetical studies to account for suspected publication bias*

**Filled (Dark/Grey) Circles: represent the actual data points collected from the existing literature (observed studies). Open (White) Circles: These represent the hypothetical studies to create symmetry.*

Prevalence of DM in LBP subgroup and non-LBP subgroup

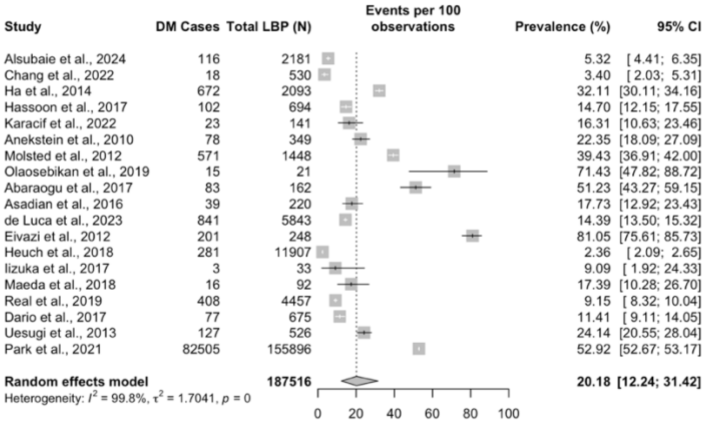

Figure 14 - *Prevalence calculations with 95% CI; DM in LBP population.*

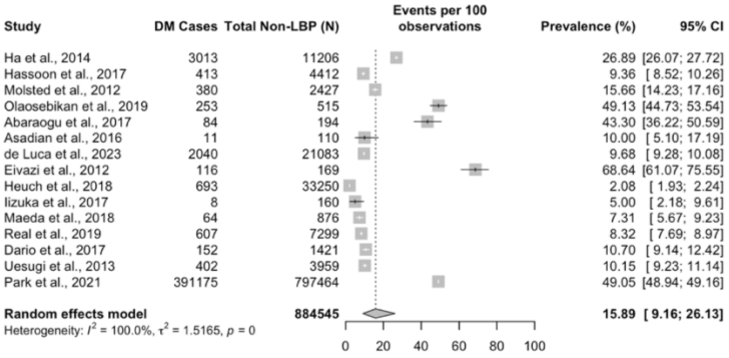

Figure 15 *– prevalence calculations with 95% CI; DM in non-LBP population.*

Comparative analysis of DM prevalence in LBP vs non-LBP and publication bias

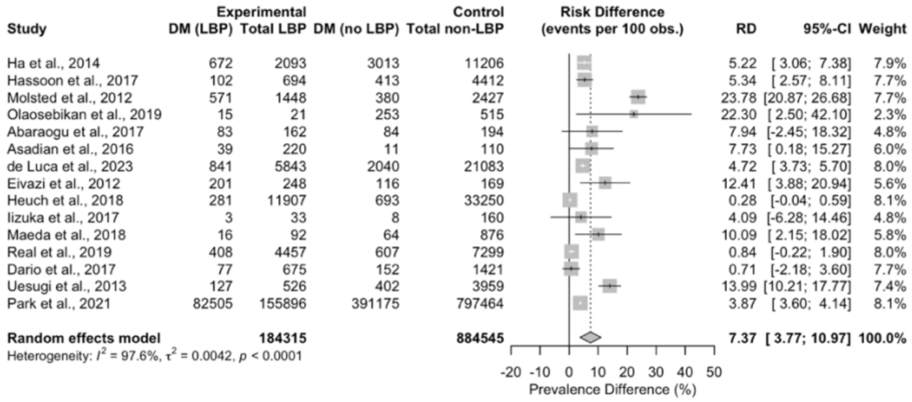

Figure 16 *– prevalence difference for DM in the LBP vs non-LBP groups (with 95% CI)*

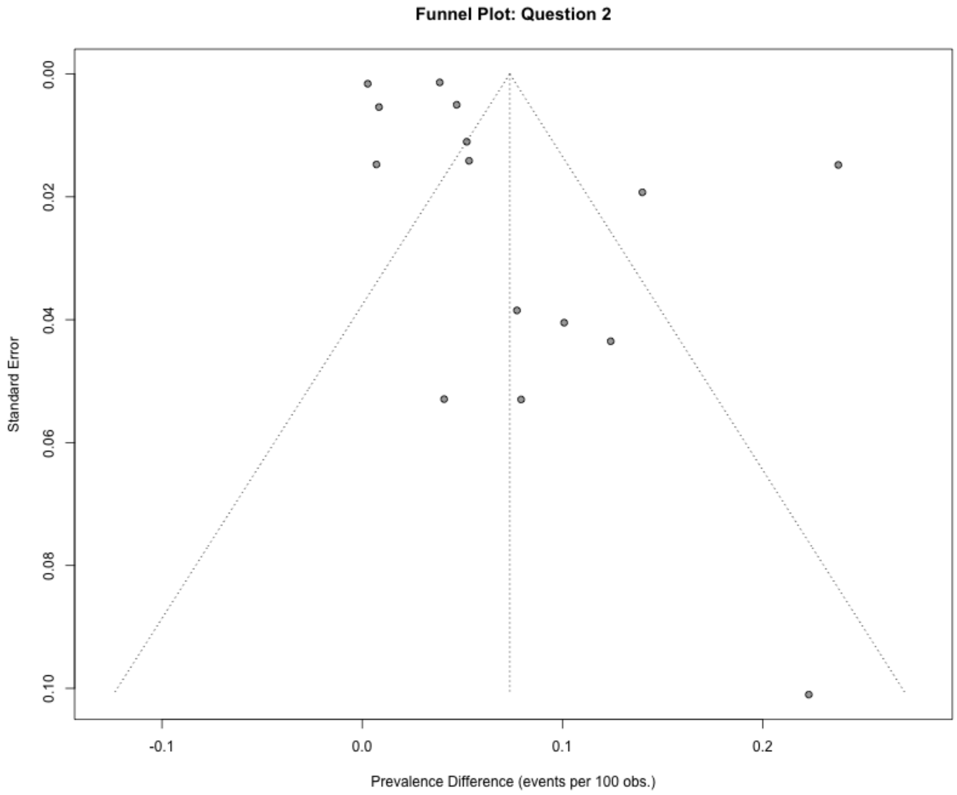

Figure 17 *– Funnel plot - prevalence difference for DM in the LBP vs non-LBP groups*

The association between DM and LBP

Publication bias

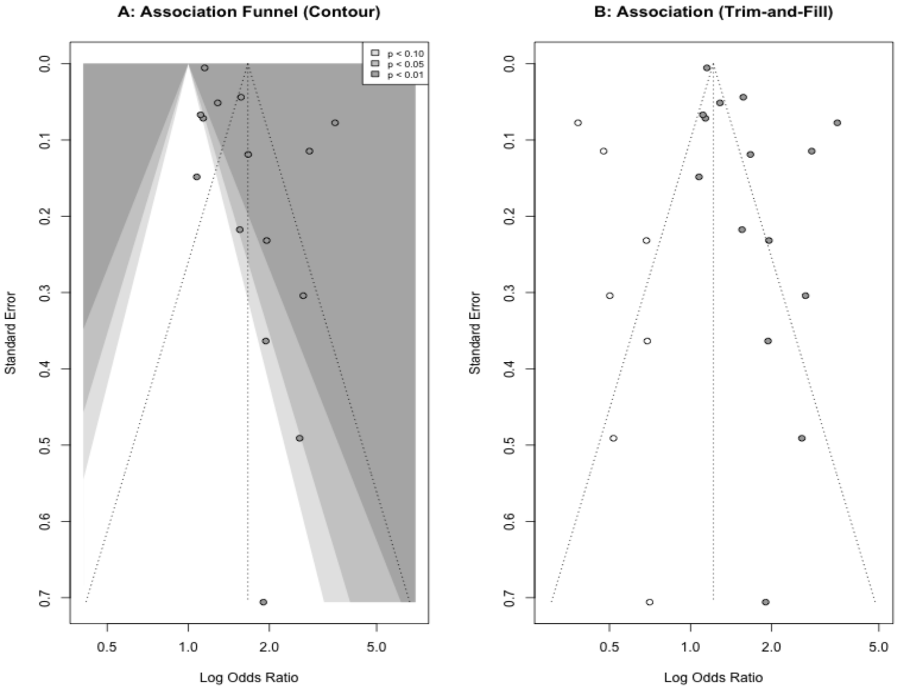

Figure 18 *- Publication bias. Assessment of Small-Study Effects: (A) The contour-enhanced funnel plot suggests that asymmetry is largely driven by studies in the significant zones. (B) A trim-and-fill sensitivity analysis was conducted to address the observed asymmetry, which simulated seven hypothetical studies to account for suspected publication bias. *Filled (Dark/Grey) Circles: represent the actual data points collected from the existing literature (observed studies). Open (White) Circles: These represent the hypothetical studies to create symmetry.*

Subgroup analysis

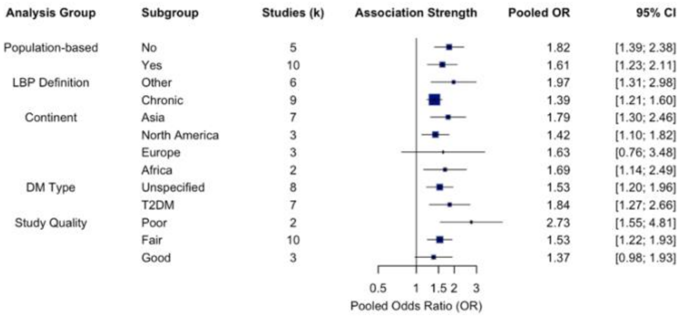

Figure 19 *- Subgroup analysis of the association between LBP and DM.* The association between DM and LBP remained consistent and statistically significant across the majority of subgroup analyses

Sensitivity analysis

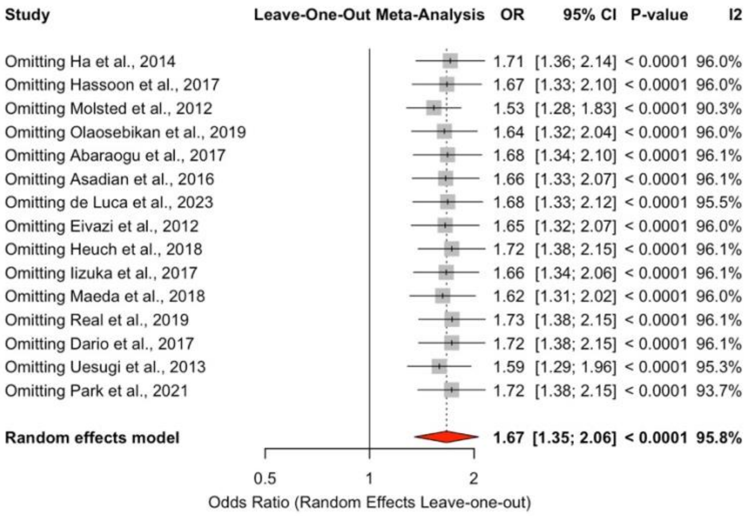

***Figure 20** - Sensitivity analysis – leave one out method.*

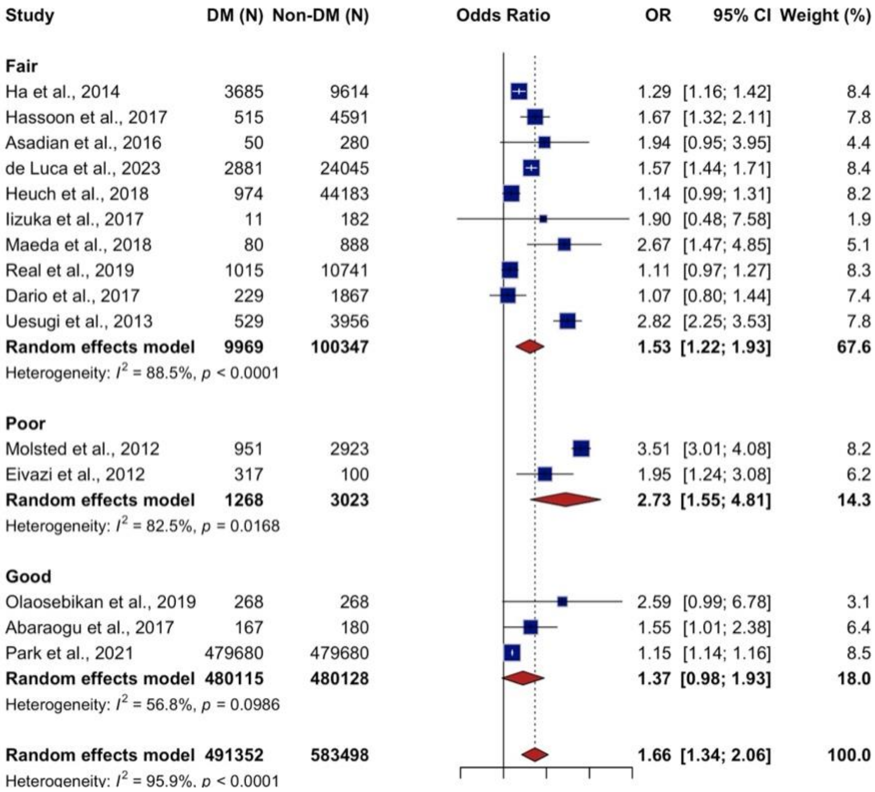

***Figure 21** - Quality-based (studies) sensitivity analysis*

Meta regression

Moderator	k	Slope (β)	p-value	R2 (Heterogeneity Explained)
Mean Age	11	+0,0375	0,033*	33,76%
Mean BMI	7	+0,0299	0,722	0,00%
Female Percentage	15	+0,0170	0,211	2,82%

***Table 6** – meta-regression of mean Age, BMI and female gender*

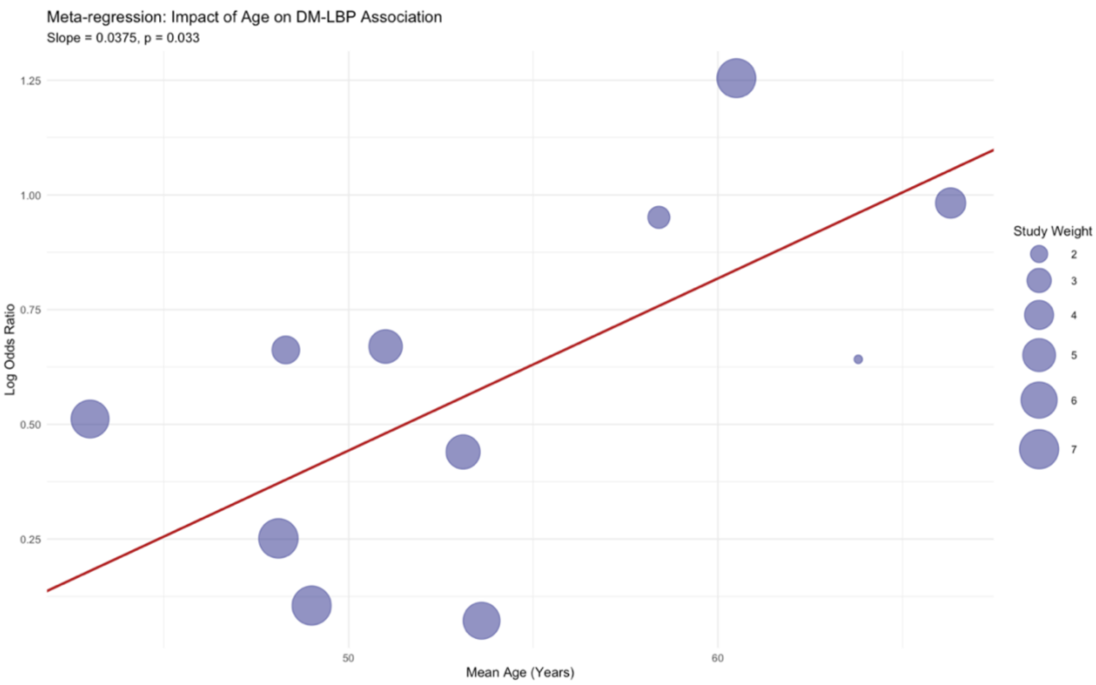

Case-Control narrative presentation

A narrative synthesis of two case-control studies complemented the longitudinal pooled analysis [52,54]. Both obtained a 'Good' quality score, though meta-analysis was precluded due to few studies retrieved and vast heterogeneity in several aspects, e.g., sample size and duration.

One of the studies examined a cohort of 345 individuals over four years[52], while the other analyzed a nationwide database (population-based) of 99,130 individuals during 17 years[54].

Both cohorts had a balanced sex distribution. Based on the available data, the lumbar spinal diagnosis was categorized as chronic LBP. Information regarding details on the diabetes definition used was incomplete and was therefore described as unspecific type.

In one of the studies[52], the prevalence of DM in males with LBP was 38,7% compared to 21.1% in the control group without LBP ($p < 0.05$). Among females, the prevalence of DM was 35.3% in the LBP group versus 26.6% in the control group without LBP. However, the difference was not statistically significant ($p = 0.218$). In males, DM was found to be a significant predicting facto for LBP, with triple the odds of development compared to their non-diabetic peers (OR 3.0; 95% CI: 1.4–6.8)[52].

In the other study[54], there were a higher likelihood of LBP diagnosis in diabetic patients compared to non-diabetic peers, OR 1.39 (95% CI: 1.36–1.43). The risk of LBP development was higher with prolonged DM exposure. The OR was 1.22 (95 % CI: 1.18–1.27) if DM was diagnosed between 50 and 65 years of age and 1.67 (95% CI: 1.56–1.79) for those diagnosed before 50 years of age. Furthermore, a higher risk of all-cause mortality was seen in individuals with DM and LBP (HR 1.36; 95% CI: 1.29–1.44).

Longitudinal association

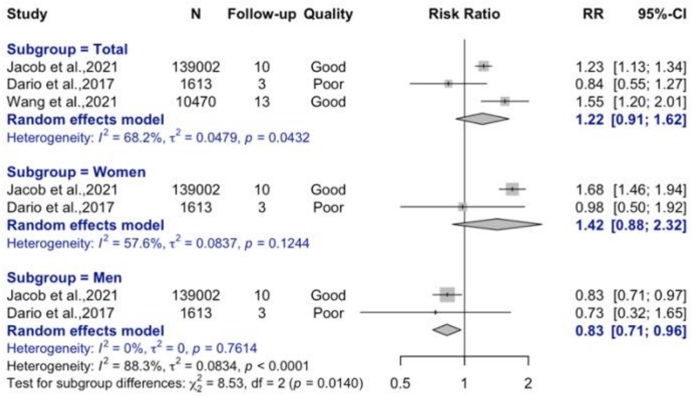

Figure 22 *- Direction DM as a predictor for LBP; Meta-analysis of three longitudinal studies including pooled sex-specific subgroup analysis*

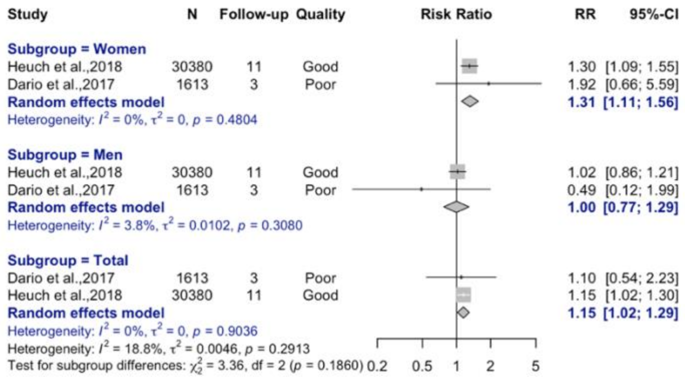

Figure 23 *- Direction LBP as a predictor for DM; Meta-analysis of two longitudinal studies including pooled sex-specific subgroup analysis*

The certainty of evidence

Outcome	Baseline	Risk of Bias	Inconsistency	Indirectness	Imprecision	Publication Bias	Final Grade	Justification
LBP in DM (Prev. Diff.)	Low (2p)	No serious concern (0p)	Serious (-1p)	Not well defined (-1p)	0p	-1	Very Low ⊕◯◯◯	Downgraded for extreme inconsistency (I2=96,1%) and evidence of publication bias (p=0,016)
DM in LBP (Prev. Diff)	Low (2p)	No serious concern (0p)	Serious (-1p)	Not well defined (-1p)	0p	0	Very Low ⊕◯◯◯	Downgraded for extreme inconsistency (I2=97,6%) and indirectness. Symmetrical funnel plot suggests no serious bias.
DM-LBP Association	Low (2p)	No serious concern (0p)	Serious (-1p)	Not well defined (-1p)	0p	-1	Very Low ⊕◯◯◯	Downgraded for serious inconsistency (I2=95,9%) and potential publication bias (p=0,018).
Longitudinal Association	Low (2p)	No serious concern (0p)	Serious (-1p)	Not well defined (-1p)	-1p	0	Very Low ⊕◯◯◯	Downgraded for imprecision (95% CI 0,91–1,62). Rated as Very Low certainty due to its observational nature and the indirectness of LBP definitions in half of the included cohort studies.

***Table** 7* – The certainty of evidence (*GRADE assessment)* for all primary outcomes
